# Unwarping GISAXS data

**DOI:** 10.1107/S2052252518012058

**Published:** 2018-10-08

**Authors:** Jiliang Liu, Kevin G. Yager

**Affiliations:** a Center for Functional Nanomaterials, Brookhaven National Laboratory, Upton, NY 11973, USA

**Keywords:** X-ray scattering, GISAXS, GTSAXS, image healing, reconstruction, distorted-wave Born approximation

## Abstract

Grazing-incidence small-angle X-ray scattering (GISAXS) is a powerful technique for measuring the nanostructure of coatings and thin films. However, GISAXS data are plagued by distortions that complicate data analysis. The detector image is a warped representation of reciprocal space because of refraction, and overlapping scattering patterns appear because of reflection. A method is presented to unwarp GISAXS data, recovering an estimate of the true undistorted scattering pattern. The method consists of first generating a guess for the structure of the reciprocal-space scattering by solving for a mutually consistent prediction from the transmission and reflection sub-components. This initial guess is then iteratively refined by fitting experimental GISAXS images at multiple incident angles, using the distorted-wave Born approximation (DWBA) to convert between reciprocal space and detector space. This method converges to a high-quality reconstruction for the undistorted scattering, as validated by comparing with grazing-transmission scattering data. This new method for unwarping GISAXS images will broaden the applicability of grazing-incidence techniques, allowing experimenters to inspect undistorted visualizations of their data and allowing a broader range of analysis methods to be applied to GI data.

## Abstract 2

Grazing-incidence X-ray-scattering (GISAXS) images contain distortions caused by refraction and multiple scattering. A new method is presented for computationally unwarping GISAXS data. The experimental data are iteratively fitted to reconstruct an estimate of the true underlying reciprocal-space scattering.

## 1. Introduction   

Grazing-incidence small-angle X-ray scattering (GISAXS) is a powerful and broadly applicable method for quantifying structural order in interfaces, coatings, thin films and multilayered nanostructures (Smilgies *et al.*, 2002 ▸; Müller-Buschbaum, 2003 ▸; Cristofolini, 2014 ▸). The scattering intensity in GISAXS is greatly enhanced because of beam projection along the sample surface (Smilgies, 2009 ▸), as well as waveguide effects that can increase the effective path-length through the sample and localize the photon field within the film (Jiang *et al.*, 2011 ▸). The geometry of GISAXS probes both in-plane (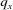) and out-of-plane (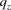) components, allowing the structure of ordered thin films to be quantified, including the material organization in the film-normal direction (Lu *et al.*, 2013 ▸). As a result, GISAXS has become an extremely popular technique for measuring three-dimensional order in thin films (Renaud *et al.*, 2009 ▸; Hexemer & Müller-Buschbaum, 2015 ▸; Müller-Buschbaum, 2016 ▸). However, experimental GISAXS detector images exhibit a number of complications because of the grazing-incidence geometry; correspondingly, data analysis is one of the main challenges in GISAXS experiments. The detector image is a (non-linearly) distorted version of reciprocal space caused by refraction of the incident X-ray beam as it enters a thin film, and refraction of scattered rays as they exit the film. Reflections of the X-ray beam off of the film and substrate interfaces will interfere with each other; the corresponding X-ray reflectivity curve modulates the observed scattering intensity. Finally, a GISAXS image has multiple superimposed scattering patterns with different apparent origins, since scattering can occur from both the incident and reflected beams. Together, these effects conspire to yield a non-trivially warped rendition of the sample’s ‘true’ reciprocal-space scattering, giving rise to the shifts and doubling effects that are characteristic of GISAXS detector images.

This data distortion is typically handled by fitting GISAXS data using a model incorporating the above-noted effects. The most popular model is the distorted-wave Born approximation (DWBA), which accounts for multiple scattering effects, such as scattering from the reflected beam (Sinha *et al.*, 1988 ▸; Lazzari, 2002 ▸; Lazzari *et al.*, 2007 ▸; Renaud *et al.*, 2009 ▸). Quasi-kinematic approximations (Heitsch *et al.*, 2010 ▸; Smilgies *et al.*, 2012 ▸) and integral potential methods (Wu, 1993 ▸, 1994 ▸) have also been described. When iteratively fitting experimental data with these methods, the GISAXS complications must be computed repeatedly, since on each iteration a candidate real-space model must be converted into reciprocal space, and then this must be warped into the experimental detector space.

Some experimental approaches have been described which circumvent the distortions of GISAXS. A thin-film sample can be measured in transmission (SAXS) geometry, with sample rotation used to progressively reconstruct the 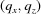 reciprocal space (Jones *et al.*, 2003 ▸). This rotational SAXS method, often called CD-SAXS since it is used to quantify a critical dimension (Hu *et al.*, 2004 ▸), has been used with great success to reconstruct lithographic (Wang *et al.*, 2007 ▸; Settens *et al.*, 2014 ▸; Sunday *et al.*, 2015 ▸), nano-imprinted (Jones, Hu *et al.*, 2006 ▸; Jones, Soles *et al.*, 2006 ▸) and self-assembled (Sunday *et al.*, 2013 ▸, 2014 ▸; Khaira *et al.*, 2017 ▸) patterns. However, this method can be time-consuming since the desired reciprocal space is probed slice by slice. An alternative is the recently described grazing-incidence transmission small-angle X-ray scattering (GTSAXS), where the X-ray beam is directed towards the downstream edge of a sample at an incident angle well above the critical angle (Lu *et al.*, 2013 ▸; Mahadevapuram *et al.*, 2013 ▸; Weidman *et al.*, 2015 ▸). This geometry allows the sub-horizon scattering to exit through the substrate with minimal absorption; in the resultant data, the refraction distortions and multiple scattering effects are nearly eliminated. While this allows a minimally distorted 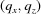 slice to be measured in a single exposure, the signal-to-noise ratio (SNR) (compared with GISAXS) is diminished because of the reduced beam projection along the sample and substrate attenuation. Moreover, this geometry requires the sample of interest to be present at the edge of the substrate, which may not always be convenient. Despite the availability of these techniques, in many cases GISAXS remains the preferred experimental technique because of the strong scattering signal that can be measured, the rapid data collection and the simplicity of the required sample and experimental protocols.

Here, we investigate whether GISAXS data can be computationally unwarped into an undistorted (SAXS-like) scattering pattern. This is a difficult inverse problem, since GISAXS data have multiple superimposed scattering contributions, all of which are convolved with distortions that change *q* positions and intensities. Moreover, this is a ill-posed mathematical problem, because of the one-to-many mapping from reciprocal space to the detector image, and the finite range of *q* space that is probed experimentally. Given the challenges in performing GISAXS data unwarping, researchers to date have instead focused on correctly approximating the effects of a GISAXS experiment, such that data can be iteratively fit using a forward model. However, there are potential advantages to unwarping GISAXS data into an estimate for the ‘true’ (undistorted) reciprocal-space scattering. Firstly, such a data representation is easier for human scientists to interpret. Common errors that are made when interpreting GISAXS data (such as mis-identifying a reflection-mode peak as a transmission-mode peak) could be avoided. Scientists can more easily identify canonical scattering patterns when unencumbered by GISAXS complications. Secondly, the speed of data fitting could be improved. When iteratively fitting GISAXS with a forward model, the grazing-incidence distortions must be computed repeatedly. If one could unwarp GISAXS data, then this undistorted scattering could be used for iterative fitting with a much lower computational cost. Thirdly, a broad range of existing scattering models (Pedersen, 1997 ▸; Förster *et al.*, 2005 ▸; Székely *et al.*, 2010 ▸; Li *et al.*, 2011 ▸; Yager, Zhang *et al.*, 2014 ▸; Senesi & Lee, 2015 ▸; Croset, 2017 ▸) and fitting software (Kline, 2006 ▸; Förster *et al.*, 2010 ▸), which are currently only intended to handle transmission SAXS data, could be used in a GISAXS context if data unwarping were available. Fourthly, a range of sophisticated data-analysis methods could be more easily applied to GISAXS data if they were remapped to an undistorted reciprocal space. For example, modern developments in correlation methods such as angular correlation analysis (Wochner *et al.*, 2009 ▸; Altarelli *et al.*, 2010 ▸; Lehmkühler *et al.*, 2014 ▸, 2018 ▸; Lhermitte *et al.*, 2017 ▸), fluctuation scattering (Chen *et al.*, 2012 ▸; Malmerberg *et al.*, 2015 ▸; Martin, 2017 ▸), or variance scattering (Yager & Majewski, 2014 ▸; Gommes, 2016 ▸) are not currently used in a GISAXS context. Finally, we note that healing data can be a useful pre-processing step (Liu *et al.*, 2017 ▸), allowing such data to be used with existing data-analysis pipelines, or input into modern machine-learning methods (Kiapour *et al.*, 2014 ▸; Wang *et al.*, 2016 ▸, 2017 ▸; Meister *et al.*, 2017 ▸).

We demonstrate that GISAXS data can be successfully unwarped to yield an estimate of the undistorted scattering. This method iteratively fits multiple input GISAXS images, using estimates of the sample’s transmission and reflectivity curves to account for refraction and multiple scattering effects. This reconstruction is found to be qualitatively correct, in general, even when the underlying assumptions are imperfect. In cases where high-quality estimates for reflectivity are available, the reconstruction is both quantitative and has a higher SNR than the corresponding GTSAXS data. Overall, the proposed method is a robust means of viewing the true reciprocal-space scattering for materials measured in GISAXS geometry.

## 2. Methods   

**Figure 1:**
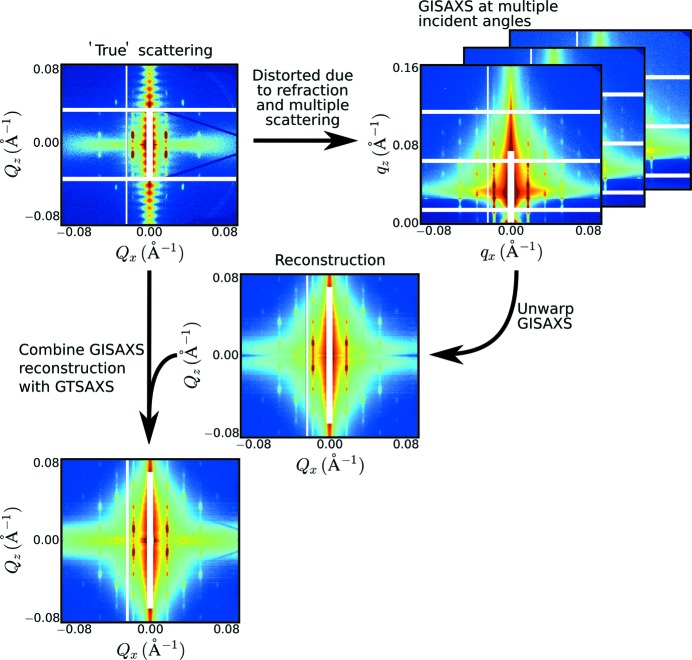
In GISAXS, the sample’s ‘true’ scattering is distorted because of refraction of incident and scattered rays, and multiple scattering effects, which lead to superimposed scattering features and non-monotonic modulation of intensities. These effects greatly complicate the analysis of GISAXS data. Here, we present a method to combine multiple GISAXS images measured at different incident angles, thereby reconstructing a faithful estimate of the unwarped reciprocal-space scattering. This reconstruction can be combined with grazing-transmission measurements (GTSAXS) if available, to further improve data quality. Example data shown in the figure were collected in GISAXS and GTSAXS geometry on a thin film of shear-aligned block copolymer. The reconstruction is difficult to assess near 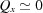 because of the beamstop. However, the structural peaks resolved elsewhere demonstrate the quality of the reconstruction.

Our reconstruction method consists of two phases. In the first phase, a highly approximate guess for the undistorted pattern is generated. As will be shown below, even an imperfect guess (even as extreme as random noise) will ultimately yield a valid reconstruction during the second phase. However, an appropriate initial guess greatly reduces the computation time in the second phase. We deploy an efficient guessing method that estimates the ratio of transmitted and reflected signals based on the mismatch between the predictions of these two signals (described in detail in Section 3). In the second phase, the initial guess is iteratively refined in an optimization loop. On each loop, a candidate undistorted scattering is converted into GISAXS data using the DWBA theory (and assumed forms for the reflection and transmission curves). By minimizing the mismatch between the computed and experimental GISAXS data, the candidate scattering pattern converges towards the ‘true’ undistorted scattering. In our implementation, we simultaneously fit multiple experimental GISAXS images (taken on the sample but at different incident angles) in order to improve coverage of reciprocal space, and compensate for untrustworthy data in any particular GISAXS image (Fig. 1 ▸). Moreover, this reconstruction can be combined with GTSAXS data (if available) to further improve data quality. While GTSAXS intrinsically records a nearly undistorted image, the proposed reconstruction takes advantage of the much stronger scattering intensity of GISAXS to improve the SNR in the data.

### 2.1. GISAXS geometry   

In conventional transmission X-ray scattering (TSAXS/TWAXS) the detector image can be directly related to reciprocal space. More precisely, for scattering with X-rays of wavelength λ, elastic scattering arises from the surface of the Ewald sphere in reciprocal space (a sphere with radius 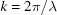). Measurements at scattering angle 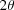 probe the sample’s reciprocal space at the coordinate 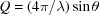; one can qualitatively say that the measured scattering arises from the slice where the Ewald sphere intersects the sample’s reciprocal space. In grazing-incidence experiments, refraction of incident and scattered rays shifts and warps reciprocal space, leading to a distorted version of the data on the detector (Toney & Brennan, 1989 ▸; Busch *et al.*, 2006 ▸; Lazzari *et al.*, 2007 ▸; Breiby *et al.*, 2008 ▸). Multiple scattering causes multiple ‘copies’ of the scattering pattern to be superimposed on the detector, with intensities modulated non-monotonically (Lee, Park, Yoon *et al.*, 2005 ▸). In this work, we use *Q* to denote coordinates in the sample’s intrinsic reciprocal space (undistorted scattering) and use *q* to denote coordinates computed from the position on the detector image. Correspondingly, we differentiate between intensity of the sample’s reciprocal-space scattering as 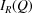, and experimentally measured intensity on the detector as 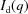.

**Figure 2:**
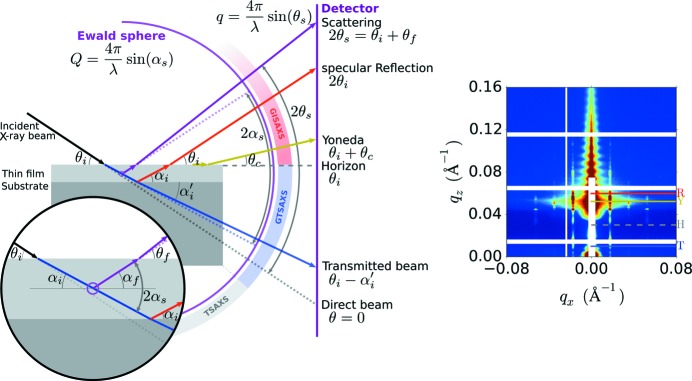
Experimental geometry for grazing-incidence scattering experiments. (Left) An incident X-ray beam (black) is refracted upon entering a thin film, and further refracted upon entering the substrate. Thus, the transmitted beam (blue) is shifted relative to the direct-beam position. Scattered rays (purple) are also refracted as they exit the film. As a result, a scattering event (purple ring) occurring with angle 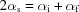 in the sample’s reciprocal space (*Q*) is observed at a different apparent scattering angle (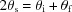) and therefore experimentally measured on the detector at a different reciprocal-space coordinate (*q*). (Right) An example GISAXS image, with the 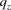 positions noted for the transmitted beam (T), the horizon (H), the Yoneda (Y) and the specular reflected beam (R).

**Figure 3:**
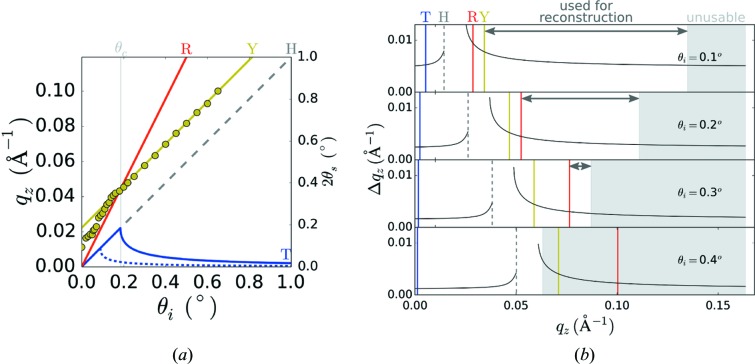
(*a*) Position along the vertical direction detector (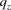) for various experimental features, as a function of grazing-incidence angle (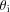), for 13.5 keV X-rays. The horizon (H, dashed gray line) is the plane of the sample itself; sub-horizon scattering must transit through the substrate (and suffer from absorption) while scattering above the horizon exits the film and travels through a vacuum. The specular reflected beam (R, red) appears at 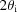. When the exit angle matches the critical angle (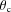) of the film or substrate, one observes enhanced intensity on the detector. This Yoneda (Y, yellow) scattering is offset from the horizon by 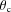. The circles are experimental data, where the position of maximum low-*q* scattering was identified as the Yoneda, for a block-copolymer thin film on a Ge-coated silicon substrate. The solid yellow line is the theoretical expectation for the critical angle for this substrate. When the incident angle is below the critical angle for the polymer film, the Yoneda shifts to this lower critical angle instead. The transmitted beam (T, blue) is shifted from the direct-beam position (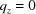) because of beam refraction (the dashed line denotes the position of the transmitted beam within the polymer layer). (*b*) The refraction distortion (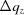) as a function of position along the detector is strongly nonlinear, and depends on both 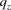 and the incident angle (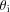). The positions of the various features are denoted by vertical lines: transmitted beam (blue), horizon (dashed gray), substrate Yoneda (yellow), and specular reflection (red). There is a gap in the data between the horizon and the film critical angle. Data close to this gap are generally unusable for reconstruction owing to the large and strongly non-linear distortion. The gray shaded region denotes the area unusable for reconstruction because of the finite range of 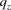 sampling (refer to *Results and discussion* [Sec sec3]); the horizontal arrow denotes the span of 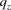 used for reconstruction.

Refraction of both the incident and scattered X-ray beams occurs owing to the different refractive indices (for X-rays) of the ambient medium (typically a vacuum), and the thin film being studied. Although for X-rays such refractive index differences are small 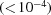, at the shallow incident angles used in GISAXS (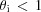
°) beam refraction becomes non-negligible (Fig. 3 quantifies the shifts of beam positions and scattering features for the typical case of a polymer film coated onto an inorganic substrate). For an incident beam impinging on a thin film at angle 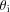 (measured with respect to the sample plane), the beam is refracted to a smaller angle of 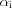 within the film (refer to Fig. 2 ▸ for a diagram of GISAXS geometry), 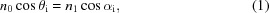
which is simply the cosine form of Snell’s law (with angles measured relative to the film plane), where 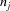 is the refractive index of the layer *j*, and we follow the convention that the ambient medium is layer 0, the thin film being studied is layer 1, and the substrate is layer 2. For X-rays, a vacuum has a refractive index of 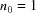, while typical materials exhibit refractive indices slightly smaller than 1; this leads to total external reflection of X-rays when the incident angle is below the critical angle 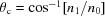. A nanostructured thin film will lead to X-ray scattering in the small-angle regime. Scattering within the film at an angle of 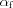 (relative to the plane of the film) will be refracted to 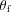 as it exits the film surface (Fig. 2 ▸ inset). We note that since the critical angle can be conveniently measured in GISAXS or X-ray reflectivity (XRR) experiments, it is useful to consider the refraction equations [such as equation (1)] in terms of 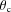, 
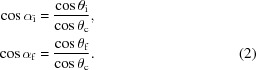
As can be seen (Fig. 2 ▸), the sample scattering of 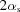 is measured experimentally at a larger angle of 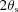. In terms of reciprocal space, scattering from 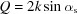 is observed at 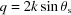 (*c.f.* Fig. 2 ▸). The shift in the position of scattering features can be quantified by (Lu *et al.*, 2013 ▸), 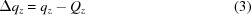


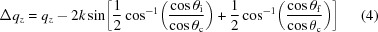


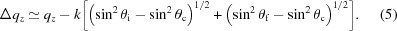
The magnitude of the refraction distortion is plotted in Fig. 3 ▸(*b*). In addition to a constant offset throughout 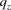 (which can be attributed to the refractive shift of the transmitted beam within the film), there are non-linear distortions that are most extreme as one approaches the thin-film critical angle (which can be attributed to refraction of the outgoing scattered ray). There is a gap in GISAXS data between the horizon and the film critical angle, since scattering within the plane of the film will be refracted out at angle 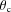 relative to the horizon.

When the exit angle for scattering matches the film critical angle (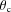), one can infer that the scattering arose from a beam propagating nominally within the film plane. Such scattering events effectively probe a greater scattering volume, leading to a substantial increase in the intensity of the measured scattering. Experimentally, one thus observes a stripe of intense scattering at the detector for angle 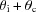 (Fig. 3 ▸ *a* and Fig. S1 in the Supporting information). This ‘Yoneda scattering’ (Yoneda, 1963 ▸; Vineyard, 1982 ▸) is useful for measuring the critical angle and thus density of the material being probed, and for enhancing otherwise weak scattering signals.

The incident beam is refracted at the ambient/film interface, and further refracted as it crosses the film/substrate interface. Thus, one measures a transmitted beam on the detector at a position slightly shifted (positive 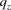) relative to the direct beam (Fig. 3 ▸ *a*). In grazing-incidence geometry, the incident beam may also be partially or fully reflected from the substrate interface (red arrows in Fig. 2 ▸). Indeed, below the substrate critical angle, the beam will be completely reflected. The specular beam is reflected from the substrate at the incident angle within the film (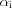) and refracted so that it exits the film at the external incident angle (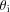). In other words, the specular reflection is measured at the expected position on the detector of 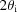 with respect to the direct beam (Fig. 3 ▸ *a*). The reflected beam, while traveling within the sample, can give rise to scattering events as well. This scattering pattern will be shifted on the detector by 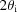 owing to the direction of the reflected beam, and by an additional amount because of the aforementioned refraction effects.

These refraction and reflection effects greatly complicate the analysis of GISAXS data, requiring more complex calculations during data fitting, and some knowledge of material properties (such as 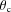). The distortions decrease rapidly (non-linearly) with increasing incident angle. For GTSAXS at sufficiently large 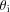, the detector image becomes a slightly shifted, linear-intensity representation of reciprocal space; *i.e.* 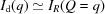. In practice, only a modest incident angle (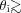 0.6°) is necessary to greatly reduce the distortions. Thus, in this work we use careful GTSAXS measurements to probe the ‘true’ scattering, and use this as a means of evaluating the quality of our reconstruction method.

### 2.2. Distorted-wave Born approximation   

For conventional SAXS data, one can typically model the data in the Born approximation, wherein one assumes that the photon field throughout the sample is uniform and simply given by the incident plane wave. In other words, the scattering of objects within the sample is assumed to be sufficiently weak (compared to the high-flux incident beam) that it does not substantially perturb the overall field. This ignores the possibility of multiple scattering, wherein the scattering from one nano-object in the sample is scattered again by another nano-object. In the case of GISAXS, this approximation breaks down. In particular, the shallow incident angles give rise to non-negligible reflection at interfaces. Thus, scattering can occur because of the incident beam, as well as the beam reflected off the substrate. The scattering itself can also be reflected. In principle, arbitrary sequences of scattering and reflection events contribute to the overall image measured on the detector. When the incident angle is close to the film critical angle, the beam can couple into waveguide modes, where the beam travels essentially within the film plane, and generates a pattern of standing waves within the film (Narayanan *et al.*, 2005 ▸; Lee *et al.*, 2006 ▸; Jiang *et al.*, 2011 ▸, 2012 ▸). This greatly enhances the effective scattering volume probed by the beam and thus the measured scattering intensity. These complex multiple-scattering effects can be accounted for by computing the total field within the sample through the interference of all possible transmission/reflection/scattering events. This computationally complex problem is typically simplified, with only the most intense scattering contributions considered. The most popular approach is the DWBA, wherein one considers the scattering from the incident field (Born approximation) as well as the first three multiple-scattering terms (Sinha *et al.*, 1988 ▸; Lazzari *et al.*, 2007 ▸; Renaud *et al.*, 2009 ▸).

**Figure 4:**
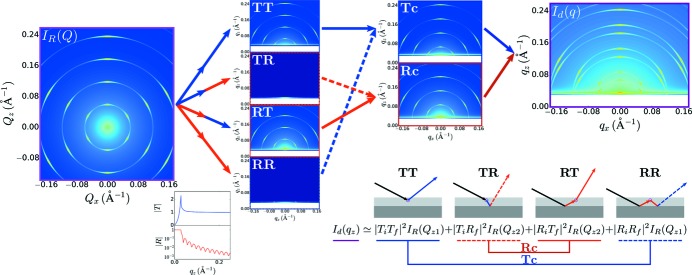
Graphical representation of the distorted-wave Born approximation (DWBA) used to calculate the total scattering intensity in a GISAXS experiment. The DWBA has four terms (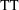, 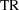, 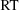 and 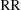) which can be understood in terms of the four most probable configurations for a scattering event. The scattering amplitude from these four terms interfere coherently; to a good approximation their intensity contributions are simply additive. The four scattering terms can be grouped into two scattering channels: one where the scattering pattern is centered about the transmitted beam (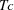 channel) and one where the scattering is centered about the reflected beam (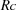 channel). The summation of these two channels yields the measured detector image [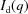], accounting for the doubling, shift and distortion of the scattering patterns observed in GISAXS.

In the DWBA, the measured scattering intensity 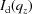 is computed using 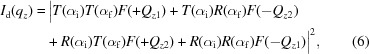
where 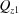 and 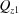 are vectors in reciprocal space that take into account the beam direction within the film, 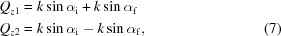
and 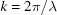. While 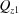 corresponds to the typical definition of reciprocal space, 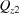 represents a shift caused by a (single) reflection event. If two reflection events occur, the reciprocal vector instead becomes 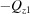. As noted previously, the 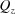 values must be computed after taking into account the refraction effect appropriate for that 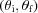. The scattering of the sample itself is captured by 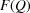, which may be thought of as the form factor and/or structure factor of the sample. The four terms in the DWBA can be interpreted physically as four possible configurations for a scattering event (Fig. 4 ▸). The first term represents scattering that occurs directly from the incident refracted beam (Born approximation). We denote this term 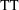 since the incident and scattered rays are transmitted (as opposed to reflected). The second term represents the case where scattering from the incident beam is reflected from the substrate interface (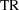). The third term represents scattering from the reflected beam (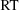). The final term covers the case where the incident beam is reflected, scattering occurs, and this scattering is itself reflected from the substrate (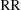). The factors of 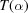 and 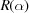 represent the amplitudes of the transmission and reflectivity factors, respectively, evaluated for the angle of the beam within the film (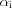 or 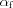). These can be computed in the usual way by applying the multilayer matrix formalism appropriate for the system being considered (Rauscher *et al.*, 1999 ▸; Renaud *et al.*, 2009 ▸) (also refer to Fig. S2 in the Supporting information).

Although the DWBA treatment of GISAXS data is necessarily an approximation, it is very successful in reproducing experimentally observed data (Lee, Yoon *et al.*, 2005 ▸; Tate *et al.*, 2006 ▸; Busch *et al.*, 2006 ▸) since the four terms it considers are the dominant sources of scattering, with additional terms involving progressively more 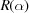 prefactors, which correspondingly decreases their intensity.

It is important to note that both the refraction effects and multiple-scattering effects of GISAXS occur in the 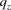 direction. Beam refraction shifts scattering along the 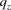 direction, and the transmission/reflection coefficients [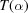 and 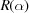] depend only on the angles within the plane of incidence (and are invariant across 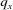 at constant 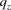). This allows us to directly equate the *x* coordinate of the detector space and the true reciprocal space (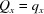), and allows different 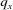 columns within the GISAXS reconstruction to be considered independently.

### 2.3. Simplified DWBA   

We investigate a series of simplifications to the DWBA formalism for two reasons. Firstly, any simplification which reduces computational complexity will allow the iterative reconstruction to converge more rapidly. Secondly, we wish to use a form of the DWBA equations that emphasizes physically measurable quantities (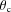, measurable aspects of reflectivity, and intensities rather than complex amplitudes) to minimize the number of additional assumptions about the sample’s structure or scattering required during reconstruction. The DWBA equation (6) can be expanded *via* multiplication to yield 16 terms, which can be regrouped as (the notation 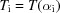 and 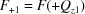 is used for compactness), 
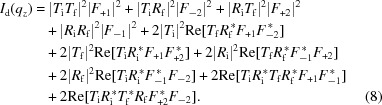
The first four terms in equation (8) can be thought of as the intensity (amplitude squared) of the four scattering configurations considered in the DWBA (the independent scattering), while the remaining terms are cross terms representing interference effects between these primary components. We note that many of these terms decrease significantly as we probe to larger 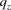 (especially any that contain a factor of 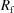). As we shall see later, our method focuses on data in this large-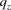 regime, and thus many of these terms can be ignored. It is also worth emphasizing several differences between the independent terms and the cross terms. While the independent terms are strictly real and positive, the cross terms are complex and may be positive or negative; in the general case, the cross terms will not be in-phase and will thus partially or fully cancel out one another. Furthermore, in a real GISAXS experiment, the beam probes an ensemble of nanostructures that give rise to an average scattering signal. As noted by Lee, Park, Yoon *et al.* (2005 ▸), the ensemble average over scatterers with uncorrelated randomness causes the average of the cross terms to be small. Thus, in many experimental scenarios, these cross terms give rise to only subtle high-frequency modulations in the data (especially near the critical angles of the film or substrate) and can be neglected in the data analysis (Omote *et al.*, 2003 ▸; Lee, Park, Hwang *et al.*, 2005 ▸) (refer to Fig. S3 in the Supporting information for an example). On the other hand, in the limit of monodisperse structures being probed by a highly coherent beam, these cross terms may substantially influence the scattering image.

Defining the scattered intensity in reciprocal space as 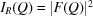 (and ignoring cross terms), equation (8) can be written as 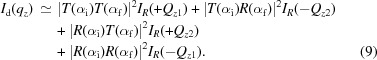
Since reciprocal space is centrosymmetric [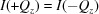], scattering patterns will exhibit twofold symmetry in the small-angle limit (although this is violated in cases of highly oriented materials). Taking advantage of this simplification, and noting that 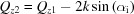, we can rearrange equation (9) into 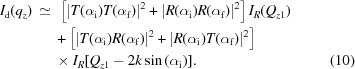
The first term of equation (10) (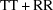) can be identified as the scattering that is centered about the transmitted beam on the detector image; we refer to this as the transmitted channel (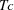). The second term (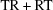) can be identified as the scattering that is shifted on the detector image (by 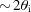) and is centered about the specular reflected beam; we refer to this as the reflection channel (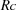). The summation of these two channels gives rise to the overall detector image (Fig. 4 ▸).

### 2.4. Iterative reconstruction   

We deploy a straightforward iterative algorithm to reconstruct the sample’s reciprocal space. We generate a candidate reconstruction 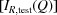, convert this to a detector image 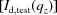 using the above-described refraction and multiple-scattering corrections {which can be thought of in terms of a transformation function 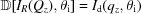}, and compare this with the experimental data. We simultaneously fit multiple GISAXS images taken at different incident angles. Thus, we compute the mismatch (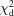) between the candidate detector image and the experimental GISAXS data 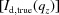 by summing the residuals across the 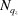 available pixels of the 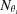 GISAXS images, 
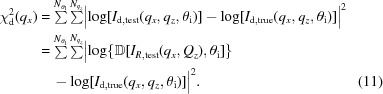
The residuals are computed on the logarithm of the data, which helps to fairly balance the contribution from intense scattering features (low-*q* diffuse scattering, sharp peaks, *etc*.) and weaker features (high-*q* scattering, higher order peaks, *etc*.). As will be described later, selecting an appropriate sub-range for 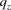 is crucial to obtaining a reliable reconstruction. Conversely, including a greater number of GISAXS images will generally improve the quality of the reconstruction. We minimize 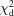 using a multi-variable constrained optimizer, which iteratively converges towards smaller values of the target function using successive local refinements. In particular, we select a bound pseudo-Newton method (Byrd *et al.*, 1995 ▸; Zhu *et al.*, 1997 ▸; Morales & Nocedal, 2011 ▸), finding it to converge more rapidly than unconstrained Newton or trust-region methods (Conn *et al.*, 2000 ▸). We terminate the iteration when 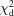 no longer improves with continued searching. This optimization procedure is repeated for each column of constant 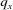 across the GISAXS image. In principle, these columns are mathematically independent and can be solved in parallel. In practice, there are advantages to exploiting the similarity in neighboring columns to improve the convergence speed.

Our method is implemented in the Python programming language (Millman & Aivazis, 2011 ▸), taking advantage of the *scipy* library for fast optimization (Oliphant, 2007 ▸), the *numpy* library for numeric handling (Walt *et al.*, 2011 ▸) and the *matplotlib* library for plotting (Hunter, 2007 ▸). The minimization is performed using the scipy.optimize.minimize function, and invoking the L-BFGS-B method.

### 2.5. Experimental data   

**Figure 5:**
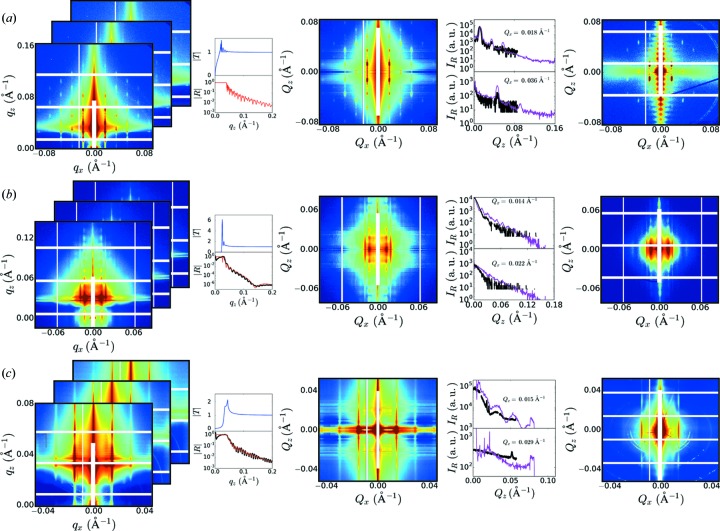
Example reconstructions based on experimental GISAXS data. Each row shows (from left to right) the set of GISAXS images, the transmission and reflectivity curves (model curves in blue/red; experimentally measured reflectivity in black), the reciprocal-space reconstruction 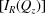, select linecuts through the reconstruction (reconstruction in purple, GTSAXS data in black) and the experimental GTSAXS image. The presented data are (*a*) a self-assembled phase of horizontally oriented cylinders that form a hexagonal lattice, (*b*) multilayered block-copolymer nanostructures and (*c*) an in-plane array of inorganic nano-dots.

To validate our methods, we conducted a series of X-ray scattering measurements on nanostructured thin films using synchrotron beamlines. Data presented here were collected at the X9 beamline of NSLS (at X-ray energy of 13.5 keV), at the Soft Matter Interfaces (SMI, 12-ID) beamline at NSLS-II (11.0 keV) and at the Complex Materials Scattering (CMS, 11-BM) beamline at NSLS-II (13.5 keV). Samples were carefully aligned with respect to the incident beam using both the direct-beam scattering intensity and the position of the reflected beam on the detector. GISAXS data were collected across a range of incident angles. Corresponding GTSAXS data were collected by increasing the incident angle and realigning the sample height such that the beam was impinging on the downstream edge of the sample, thereby maximizing the sub-horizon scattering intensity (Lu *et al.*, 2013 ▸). We selected a range of samples to study, in order to test the performance of our method on different types of scattering images, taking advantage of both lithographic methods, which can generate extremely precise nanostructures (Luttge, 2009 ▸), and block-copolymer self-assembly, which can be used to generate a variety of two-dimensional and three-dimensional morphologies over wide areas (Doerk & Yager, 2017 ▸). Lithographic line-gratings fabricated using electron-beam lithography were measured with the X-ray beam along the grating groove length (Johnston *et al.*, 2014 ▸). Three-dimensional polymer nanostructures were fabricated using a commercial two-photon absorption laser lithography tool (Nanoscribe Photonic Professional GT). Thin films of a block-copolymer-cylinder phase, with cylinder axes oriented perpendicular to the film normal were shear-aligned using photo-thermal methods (Majewski & Yager, 2015*a* ▸,*b* ▸; Majewski, Rahman *et al.*, 2015 ▸) and measured with the X-ray beam along the shear direction (*cf.* Figs. 1 ▸, 2 ▸, 3 ▸ and 5 ▸ *a*). Multilayered nanostructures were fabricated by iterative assembly of block-copolymer phases combined with sequential infiltration synthesis to convert the self-assembled organic phases into inorganic replicas (Rahman *et al.*, 2016 ▸) (Figs. 5 ▸ *b* and 6). An ordered and aligned array of hexagonally packed nano-dots, fabricated using block-copolymer self-assembly was also measured (Fig. 5 ▸ *c*) (Choo *et al.*, 2017 ▸). To compute the transmission and reflection coefficients (*T* and *R*) for these samples, we either estimated the layer profile from knowledge of sample fabrication, or experimentally measured the X-ray reflectivity (XRR) curve (Rigaku Ultima III), and used the best fit to compute the coefficients. For instance, for the sample shown in Fig. 5 ▸(*a*) (data collected for 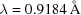) we use a model with a 125 nm polymer layer (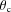 = 0.09°), on a 100 nm Ge layer (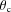 = 0.17°), on a 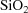 substrate (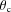 = 0.14°). For some tests, we generated synthetic data (Yager *et al.*, 2017 ▸), allowing us to systematically vary properties of reciprocal space and the film structure. For the synthetic data used throughout the paper (such as Fig. 8), we assume a 30 nm polymer layer (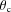 = 0.11°), on a 100 nm 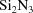 layer (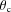 = 0.09°), on a Ge substrate (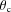 = 0.20°).

## 3. Results and discussion   

Although the transformation from SAXS data to GISAXS data is well understood (see Section 2), inverting this transformation is not readily possible. The inversion is a mathematically ill-posed problem, owing to experimental noise, the one-to-many mapping between the 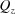 and 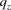 spaces, and the limited range of the data. A scattering feature at a particular 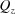 will contribute intensity to two different 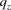 locations (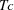 and 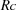 channels). Similarly, in the inverse problem, the intensity at a particular 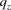 has contributions from two different 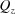. Reconstructing 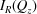 thus requires solving a coupled set of equations; however, the limited range of experimentally available 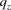 makes the problem formally underdefined (with references outside the available data range). On the other hand, the inversion is only weakly underdefined, and an approximate solution can be found using modest, physically reasonable assumptions. Our method for reconstructing the true reciprocal-space scattering image consists of first constructing a reasonable guess for 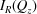, and then iteratively adjusting this estimate by fitting available GISAXS data 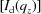, minimizing the fit-error [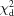, equation (11)]. Reconstruction involves repeatedly applying the DWBA transformation (Fig. 4 ▸), and thus requires knowledge of 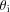 for the GISAXS data, and reasonable estimates for 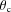, 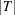, and 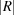. The latter can be estimated from knowledge of the sample makeup, or measured using X-ray reflectivity.

Only a subset of a GISAXS image contains data usable for the reconstruction. While the finite size of the X-ray detector provides a hard limit on the 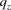 range, other effects impose additional constraints. At high 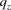, the signal becomes weak and unreliable. At small 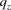 (approaching the film or substrate critical angle), the refraction distortion becomes large and highly nonlinear, making the mapping to 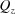 error prone. In this regime, intensity corrections also become more error prone (sensitive to the assumed form of 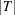 and 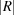). In practice, we select only the GISAXS data that are strictly above both the substrate critical angle and the reflected-beam position (yellow and red lines in Fig. 3 ▸). The 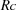 channel is symmetric about the reflected-beam position, and thus data below the reflected beam are largely redundant. The reconstruction method is implicitly identifying a form of 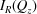 that yields 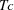 and 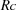 channel predictions mutually consistent with the data. This procedure is only well defined over the 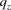 range where the two channels overlap. Since the 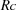 channel is shifted by 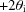 relative to the 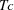 channel, any data within 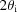 of the upper edge of the detector (gray shaded region in Fig. 3 ▸) are not usable (overlap condition is not satisfied).

The GISAXS incident angle influences the reconstruction quality. For large 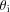, the overlap region becomes very small and the corresponding 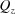 range of the reconstruction is limited. In principle, large 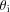 should simplify the reconstruction since the reflection components become weak, and one need only consider the Born-approximation term of the DWBA. However, the concomitant decrease in overall scattering intensity makes the data noisier. A key feature of our approach is to simultaneously fit a set of GISAXS images at different 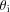 (rather than a single image). This provides some redundancy in the data, which helps to average out noise in the data and artifacts from the fitting procedure, and to converge to a unique minimum during reconstruction. This is especially important since scattering features are shifted because of refraction, and convolved with the transmission and reflectivity curves. At a particular incident angle, two distinct scattering features may overlap, or a scattering feature may be suppressed (*e.g.* because of a minimum in 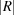). By combining multiple incident angles, each feature in 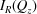 is cleanly represented somewhere in the overall GISAXS data set. The selection of incident angles of course influences the quality of the reconstruction. Selecting similar 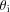 is sub-optimal since the GISAXS curves are similar and thus do not offer meaningfully independent constraints. On the other hand, selecting very large 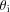 is not helpful since such data have a very limited usable 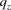 range. It is also important to note that the computation-time scales linearly with the number of 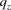 data points, and thus with the number of 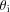 one considers. We empirically find that selecting three GISAXS images, with incident angles spaced by 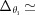 0.02° yields robust reconstructions (*e.g.* 0.14, 0.16, and 0.18°). An additional benefit to using multiple incident angles is that this implicitly fills any gaps in the data caused by, for example, inter-module gaps in the detector. Because each 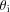 probes a slightly different part of 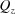 space, the gaps are not aligned and thus a set of GISAXS images can be used to generate an uninterrupted span of 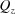. Any gaps remaining in 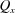 can also be filled using previously reported image-healing methods (Liu *et al.*, 2017 ▸).

**Figure 6:**
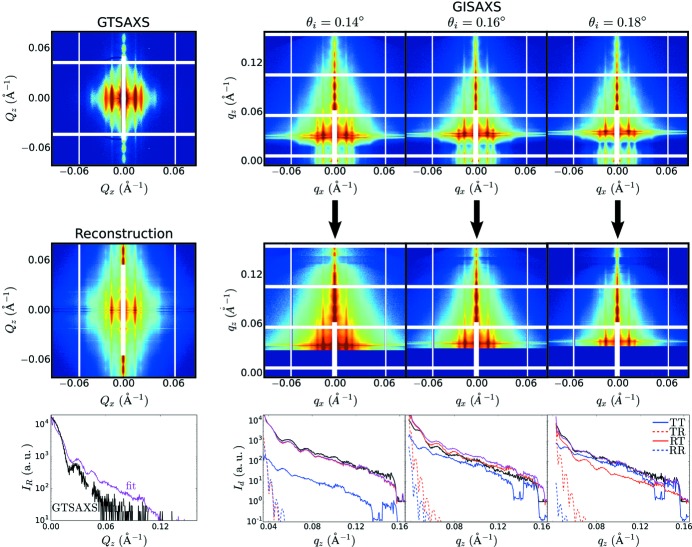
Example reconstruction of true (SAXS) scattering data based on combined fitting of multiple experimental GISAXS images. The corresponding experimental GTSAXS data are presented as an alternative estimate for the true SAXS scattering pattern (upper left). The upper row shows the experimental GISAXS images (at multiple incident angles), while the middle row shows the corresponding GISAXS fits obtained from reconstruction. The bottom row shows example one-dimensional linecuts through the data (at *q_x_* = 0.0138 Å^−1^). The experimental data are shown in black, the reconstruction/fit is shown in purple and the individual DWBA components are shown in blue or red. The direct comparison between the reconstruction and the GTSAXS data (bottom left) demonstrates that the GISAXS reconstruction is robust and has a higher SNR (GTSAXS curve, black, was offset vertically to aid in comparison).

Fig. 6 ▸ shows an example of fitting an experimental set of three GISAXS images. The fitting generates a set of GISAXS images that closely match the experimental data. The underlying 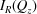 reconstruction is a close match to the true undistorted scattering (which can be estimated from the experimentally measured GTSAXS pattern). A direct comparison between the reconstruction and the GTSAXS data (Fig. 6 ▸, lower-left) highlights that the reconstruction reproduces the correct positions and scaling of scattering features (*e.g.* peaks). Moreover, the SNR of the reconstruction is much better than that available for GTSAXS (*e.g.* higher order peaks are visible), since it takes advantage of the GISAXS intensity-enhancement effects (especially beam projection over the sample surface).

A crucial aspect for rapid convergence of the reconstruction method is the selection of the initial guess for 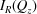. We find that the iterative reconstruction is well behaved in the sense that any initial guess will converge to the same (correct) reconstruction. For instance, initializing 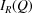 to noise, or starting with 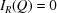 will eventually converge to a valid reconstruction (see Fig. S4 in the Supporting information). It is also worth noting that the fit-error to the experimental data (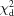) is highly correlated to the mismatch of the reconstruction (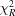); in other words, minimizing the fit-error does converge to the true reciprocal-space scattering (see Figs. S5 and S6 in the Supporting information). Although the final reconstruction quality is robustly insensitive to the starting guess for 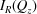, the number of iterations required for a high-quality reconstruction depends strongly on the initial guess. Thus, there is a huge computational benefit to selecting an appropriate initialization. A straightforward initialization is to simply copy the GISAXS curve into 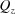 space. While this is necessarily incorrect in many ways (*e.g.* exhibiting double peaks), it at least sets a roughly correct scale for the scattering intensity (capturing the experimentally observed decay with 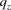). Since each 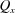 column can be reconstructed separately, another option is to select a neighboring reconstructed column as the initial guess for a new column. As might be expected, this greatly improves convergence if one selects a nearby neighbor, and is somewhat less successful if the column is far away or the scattering intensity varies dramatically along 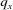 (see Fig. S4 in the Supporting information).

**Figure 7:**
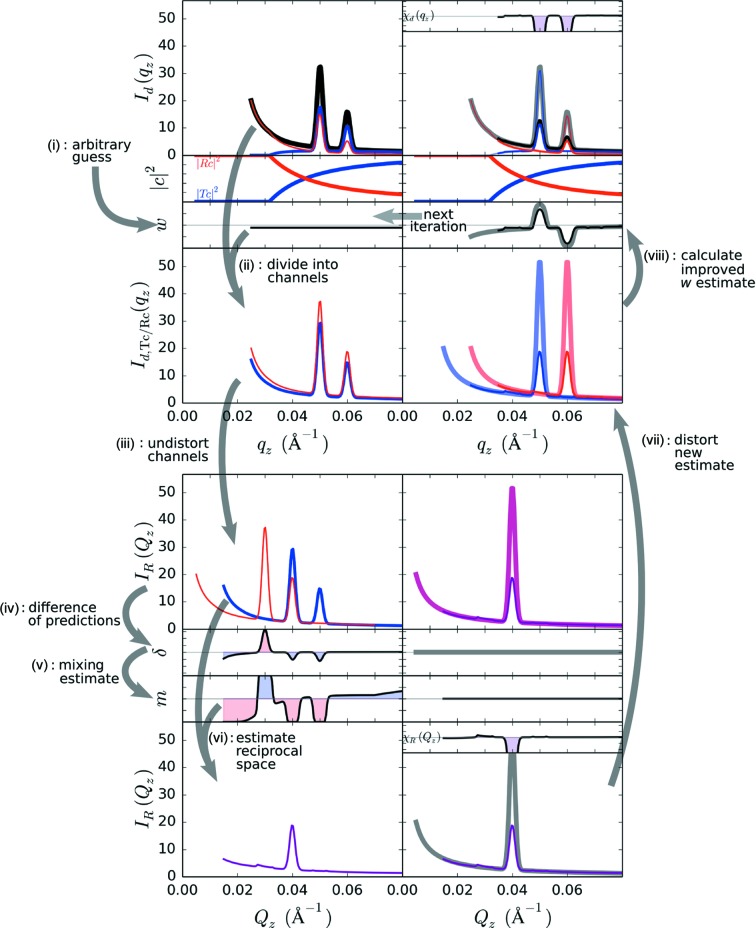
Algorithm for generating an initial estimate of the undistorted scattering 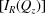 from experimental GISAXS data 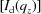. The experimental intensity is first divided into contributions from the transmitted and reflected channels (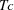 and 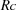) based on known transmission/reflectivity curves (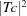 and 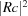) and an arbitrary guess for the ratio between the channels (*w*). The two channels are both undistorted into reciprocal space, which provides two predictions for the true scattering (which should agree). The difference between these predictions is used to compute an improved estimate for 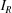. This 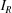 estimate can be distorted to yield new estimates for the contributions from the two channels, which provides an improved estimate for *w*. This new *w* estimate can be fed back as an improved initial guess. (Thick faded lines show true scattering contributions, while χ shows the corresponding residuals; these are of course not known during reconstruction of experimental data.) By iterating through this procedure, the algorithm converges towards a self-consistent prediction for 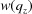 and 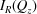.

We can generate a much better initial guess by taking advantage of the fact that a GISAXS curve conceptually contains two copies of the underlying scattering pattern because of the contributions of the 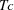 and 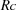 channels. We reformulate the problem of estimating the individual channel intensities [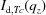 and 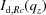] into a problem of estimating the ratio of these contributions, 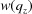. This simplifies the problem since one can estimate 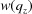 by evaluating the mismatch between predictions for 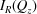 obtained from 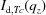 and 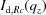 separately. The experimental data 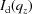 result from the summation of two contributions, 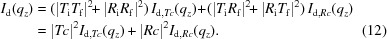
We define the ratio between the channels to be 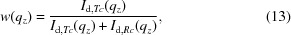
such that one can compute the two components from 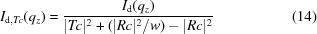
and 
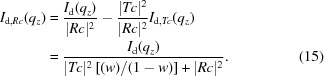
Focusing on solving for 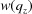 conceptually simplifies the problem. In particular, if 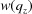 is known, one can easily compute the contributions from the two channels and can thus reconstruct the correct 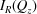 from either of these; conversely, one can estimate 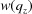 by comparing the mismatch between predictions obtained from 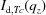 and 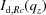, separately. An algorithm for initializing 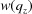 to a reasonable estimate is shown in Fig. 7 ▸. The steps are as follows: (i) we begin by arbitrarily selecting a guess for 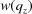. The simplest starting point is to assume equal contributions from both channels *i.e.* 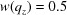. We find empirically that using 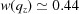 is slightly better, since in most cases 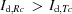, since the 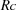 channel is shifted to higher 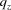 than the 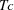 channel. (ii) Based on the assumed 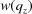, we divide the experimental 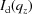 into contributions from the 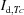 and 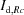 channels [equations (14) and (15)]. (iii) Based on knowledge of the refraction distortion, the two channels can be shifted from the detector space 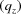 into reciprocal space 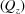. These act as two related predictions for the undistorted scattering 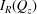. These two curves should be identical; any mismatch between them thus represents an error in our current guess of 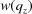. (iv) We compute the difference between our two predictions, 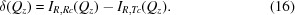
(v) From the difference between the curves, we compute a local mixing ratio 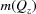, 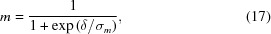
where 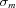 controls the character of the mixing. (vi) The purpose of 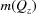 is to regulate how we mix together the two channel predictions into an improved estimate for 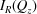. In particular, 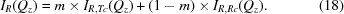
For 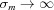, 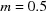, and we are thus averaging together the two channel predictions. For 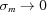, *m* becomes a step function, and we are thus thresholding, and selecting whichever curve has the lowest value (at each 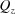). Intermediate values of 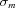 represent an intermediate strategy, where we include contributions from both curves but emphasize the curve with lower intensity. The underlying rationale is that any mismatch between the two predictions represents a mistake in our guess of *w*. In particular, if a feature (*e.g.* peak) appears only in one of the channel predictions, then it is erroneous (and can be suppressed *via m*); whereas features that appear in both channels are considered correct and will be preserved by *m*. (vii) The current estimate for 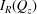 is distorted back into the detector space (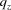). (viii) A new estimate for 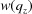 is calculated [equation (13)]. This is used as a new input [step (ii)] for another iteration of this strategy.

On each iteration, this algorithm produces an improved estimate for 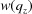. Even a single iteration of this method already yields a reasonable guess for the structure of reciprocal space (Fig. 7 ▸). The quality of this guess improves rapidly with additional iterations (see Figs. S7 and S8 in the supporting information), since the algorithm specifically focuses on changing the estimate in any regions where the two channels disagree. It is worth noting that the algorithm does not, at first, correctly predict the overall intensity of scattering features (*e.g.* peaks). However, because the method converts from intensity space 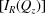 to a ratio representation 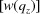, it immediately shifts back to experimentally validated intensities. This method can be applied (in parallel) to the columns of a two-dimensional data set, allowing a GISAXS image to be converted into a corresponding SAXS image in a very small number of iterations (see Fig. S9 in the supporting information).

Through a series of empirical tests, we find that the *w*-initialization method produces a reasonable reconstruction of reciprocal space for essentially any form of 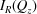. We investigated well separated sharp peaks, scattering signals with many overlapping peaks, overlapping broad peaks, diffuse scattering, power-law backgrounds, and combinations thereof; we found acceptable reconstructions in all cases. We note that, in general, this method introduces small artifacts (intensity jumps) at locations that are influenced by the overall *q* range of the data. Minor artifacts also persist at *q* values where sharp features (*e.g.* peaks) initially appeared in the ‘wrong’ channel. These artifacts cannot be completely eliminated using the *w*-initialization method, but are removed once the curve is iteratively improved by directly fitting 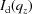. We empirically find that five to ten iterations is sufficient to obtain a reasonable *w* estimate, with additional iterations not yielding improvements in 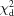. The exact rate of convergence is influenced by the *m* mixing function. We find that larger values of 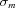 are somewhat better for scattering data with broad/diffuse features (*i.e.* averaging channel estimates is preferable) while smaller 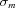 is preferable for sharp features (*i.e.* thresholding channel estimates is preferable). Moreover, there is an advantage to shifting gradually towards smaller 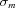 as iteration continues, which conceptually means shifting from averaging to thresholding. We select values of 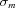 using the scale of the δ values themselves. In particular, we define 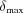 as the maximum (absolute) δ value, and find that a value of 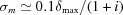 (where *i* is the iteration number) works in the general case (with 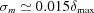 being better for sharp features, and 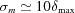 for diffuse features). Overall, the *w*-initialization strategy is a simple and robust means of obtaining a reasonable initial estimate for the structure of 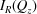 in a computationally simple manner. This initial guess is then input into the iterative reconstruction, which then refines this estimate so that it closely matches the experimental 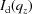.

The *w*-initialization strategy, combined with iterative fitting of GISAXS data, is able to robustly reconstruct undistorted scattering from a wide range of experimental GISAXS data (see examples in Fig. 5 ▸ and Fig. S10 in the Supporting information). The reconstruction resolves many subtle features (such as inter-peak oscillations) that are smeared or indistinct in the GISAXS data. In a data-visualization context (Zhong *et al.*, 2018 ▸), it also removes the ambiguity of deciding whether a GISAXS peak position should be interpreted with respect to the transmitted or reflected beam. Seemingly complex GISAXS patterns become much simpler to interpret after unwarping. The reconstruction method leverages the intensity enhancements of GISAXS to yield a high SNR SAXS image. When comparing with corresponding experimental GTSAXS data, it is clear that the unwarped GISAXS data resolve many features (such as weak higher order peaks) that are lost in GTSAXS because of beam attenuation through the substrate. On the other hand, the reconstructions sometimes exhibit intensity artifacts. For instance, the intensity near 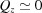 is not reconstructed faithfully, since this data comes from the error-prone portion of the GISAXS image (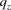 close to substrate critical angle). Where both GISAXS and GTSAXS data are available, the two can be combined (Fig. 1 ▸), wherein one can leverage both the high SNR of GISAXS data (for large 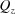) and the cleaner measurement of GTSAXS (for small 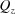).

**Figure 8:**
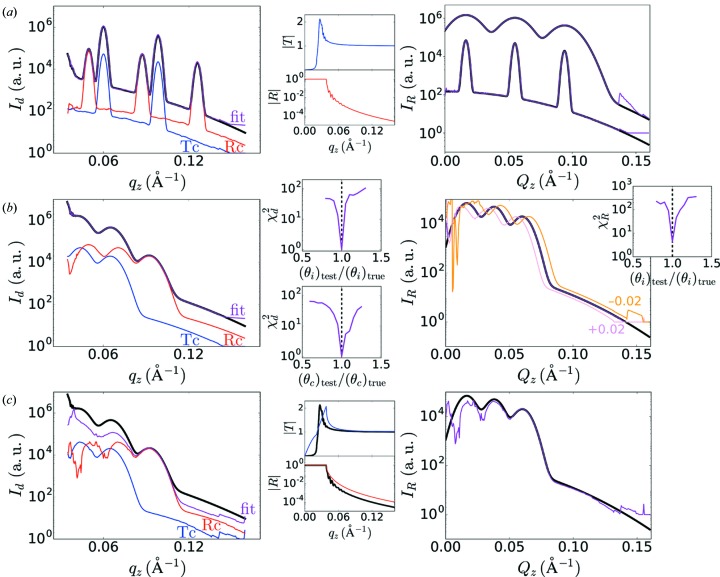
Sensitivity of reconstruction method, evaluated using synthetic data. (*a*) The input synthetic GISAXS data (black curve), 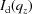 (example on left) can be iteratively fitted (purple curve), from which we reconstruct the undistorted scattering [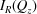, right]; the corresponding transmission channel (blue 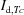) and reflection channel (red 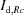) components are calculated using the known transmission (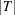) and reflectivity (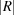) curves (shown in center). Two different examples (right) are shown (reconstruction shown in purple, true scattering in black), demonstrating that the method works for sharp and diffuse features (with artifacts appearing towards the edges of the available 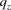 range). (*b*) The method is relativity robust to errors in the assumed incident angle (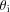) and film critical angle (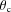). For a typical data set (left) with intentional errors introduced into 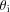, the reconstruction becomes correspondingly shifted (right, numbers indicate intentional error in 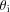). The fit-error (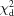) with respect to errors in the angles has a deep minimum at the true value, allowing this value to also be iteratively refined. (*c*) Errors in the assumed transmission and/or reflectivity curves necessarily corrupt the reconstruction. However, for modest errors in these curves, the reconstruction remains qualitatively correct. In the example presented here, incorrect transmission and reflectivity curves were used intentionally (center; true curves in black). The resulting reconstruction (right) exhibits artifacts yet nevertheless maintains the correct overall shape and intensity.

The reconstruction method works across a wide range of different scattering patterns, and is relatively insensitive to the shape of the reciprocal-space scattering. We evaluated the performance of our method using synthetic data (Fig. 8 ▸), allowing us to systematically compare the reconstruction to the correct scattering. Both sharp and broad features in scattering data are properly reconstructed (Fig. 8 ▸ *a*), with sharp features being somewhat easier to fit (see Fig. S6 in the Supporting information). Since the reconstruction method requires knowledge of several experimental and materials parameters, it is natural to ask whether the method is sensitive to errors in these inputs. Any error in the assumed value of 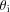 will naturally shift the reconstructed 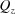 by that amount (Fig. 8 ▸ *b*), and may introduce additional intensity artifacts. We also find that an erroneous 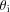 sharply increases 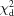. Thus, the value of 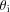 could also be refined during the reconstruction. We similarly observe that errors in 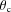 will shift the reconstruction along 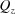 (see Fig. S11 in the Supporting information); this angle value itself can be refined since the correct value is a deep and well defined minimum in the 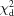 error surface. While 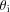 is typically precisely known from the experimental alignment, 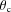 is materials and sample dependent, and should ideally be measured from the data rather than assumed. The reconstruction method requires estimates for the transmission and reflectivity curves (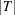 and 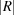). The X-ray-reflectivity curve can be measured experimentally, or estimated based on the known layering of the sample. Although the reconstruction quality obviously depends upon these curves, we find that the reconstruction is surprisingly robust to errors in these curves. For modest errors in the assumed curves, the reconstruction remains qualitatively correct (Fig. 8 ▸ *c*). This suggests that as long as a reasonable estimate for the X-ray reflectivity curve can be provided by the experimenter, the reconstruction method can yield a useful estimate for the structure of reciprocal space. With respect to the materials-layer model that underlies the reflectivity curve, we find that the reconstruction is quite robust. Errors in the film thickness shift the overall 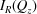 intensity (see Fig. S12 in the Supporting information) while correctly capturing the position of features; in extreme cases an erroneous thickness estimate can introduce spurious oscillations into the reconstruction. Errors in the film roughness or film absorbance (the imaginary part of refractive index) lead to only very minor deviations in the final reconstruction (see Figs. S13 and S14 in the Supporting information).

The iterative reconstruction method we employ can be computationally expensive since the method must repeatedly perform the DWBA calculations and must refine the intensity for each point in the 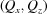 space one is constructing. The computation time increases linearly with the number of detector pixels and the number of 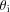 one includes. However, there are a number of ways to minimize the computational burden. Many intermediate results [distortion shift of equation (3), channel prefactors in equation (10)] can be computed once and used repeatedly. We also greatly minimize the number of iterations required during reconstruction by first generating a high-quality initial guess for reciprocal space; by using either a *w*-initialization algorithm, or using nearby reconstructed data. The 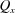 columns can be refined independently, and it is thus trivial to parallelize in this manner. We find that such a parallelization provides nearly linear speedup (*e.g.* a 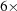 speedup when computing using eight CPU cores). The exact computation time depends on the size of the data set and the hardware being used. As a guide, we find that when fitting multiple (three) high-resolution (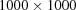 pixels) GISAXS detector images the reconstruction takes 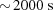 using a single modern CPU and the near-neighbor initialization strategy. Reconstructing the same image using eight CPU cores and the *w*-initialization strategy requires 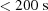. The reconstruction time could be further reduced in a number of ways. Smaller images will, of course, be even faster to reconstruct; that is, there is an advantage to only reconstructing the desired region of interest. The required set of computations is sufficiently simple that this method could also be greatly accelerated using GPUs (graphics processing units). One could also take advantage of the relative smoothness and continuity of reciprocal space, in both the 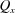 and 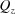 directions, by first performing a coarse-grained reconstruction, and then using this as an initial guess to a finer-grained reconstruction.

A key limitation of the method we have proposed is that one must have reasonable estimates for the film makeup. While some values (especially 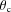) can be easily measured from the input GISAXS data themselves, other parameters (the reflectivity curve, 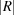) may not be easy to estimate. In the general case, a material of interest can have an arbitrary density profile in the film normal direction, with a correspondingly complex transmission and reflectivity curve. In such cases, the reconstruction will be valid only if one accurately models the layer profile. On the other hand, a great many experiments are performed on thin films that are relatively uniform. If the scattering contrast from nanostructures within the film is much smaller than the scattering contrast between the film and the ambient medium, then the reflectivity curve will be dominated by the thickness and average density of the film. In other words, for a great many GISAXS experiments, an adequate estimate for the reflectivity curve can be obtained by simply knowing the film thickness and average composition.

Although our present implementation is highly successful in reconstruction of an estimate for the undistorted scattering, there are many improved variants one could consider. As previously noted, a straightforward extension of the present method would be to include other parameters in the iterative reconstruction. Our results demonstrate that 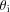 and 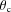 can be robustly fitted as part of the reconstruction. Some aspects of the reflectivity curve (especially the film thickness) could also be fitted as part of the 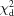 minimization. A more challenging case would be to attempt to fit the entire X-ray reflectivity curve during reconstruction; or, equivalently, to develop a model of the film’s density profile. In principle, such information is encoded within the GISAXS image, and iterative reconstruction could simultaneously extract estimates for 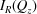 and 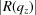 that match the GISAXS data. In our current implementation of the reconstruction, we take advantage of several approximations, which simplify the formalism and reduce computation time. However, this limits the generality of the method. In particular, we expect the method to fail when materials have layer profiles that deviate strongly from the uniform-film approximation, and when the nanostructures within the film are near-perfectly ordered over large in-plane distances. An avenue for future improvement of our method is to be able to handle these cases. To correctly reconstruct materials with complex layer profiles, one would need to use DWBA variants that include graded or multi-layered profiles for the film (Boer, 1996 ▸; Sentenac & Greffet, 1998 ▸; Lazzari *et al.*, 2007 ▸; Renaud *et al.*, 2009 ▸). This allows one to account for the intensity of transmitted and reflected components throughout the entirety of the film depth; however, in such a case one would need to have an explicit model for the real-space structure of the sample in order to account for which features in reciprocal space arise from which depths within the film. Similarly, an improved reconstruction could be achieved if one included the contribution from the cross terms of equation (8). Correct calculation of these terms requires knowledge of the complex amplitude 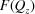, which could also be carried out using an explicit real-space model.

## 4. Summary   

We have demonstrated a method for unwarping GISAXS data, by iteratively reconstructing the true (undistorted) reciprocal-space scattering (*i.e.* a SAXS-like scattering pattern) such that it is consistent with the experimental GISAXS. We simultaneously fitted multiple GISAXS images to improve robustness, and filled any regions of reciprocal space not covered by a single image. We exploited a simplified version of the DWBA formalism to compute GISAXS images from reciprocal space. We found that despite several simplifications, and the use of assumed forms of the transmission and reflectivity curves, we are able to reconstruct reasonable estimates of undistorted scattering for a wide variety of sample structures. Overall, this unwarping method should prove to be a useful and generic method for inspecting GISAXS data, allowing experimenters to view data unencumbered by refraction distortion and multiple scattering, and in some cases yield reconstructed data of sufficient quality for rigorous analysis and fitting.

## Supplementary Material

Further details and analysis including supporting figures. DOI: 10.1107/S2052252518012058/hf5364sup1.pdf
