# Supplementary material for: Unwarping GISAXS data
Source: IUCrJ. 2018 Oct 8;5(Pt 6):737–52. doi: 10.1107/S2052252518012058 (PMC6211535; doi:10.1107/S2052252518012058)
Supplement: Supplementary file 1 [file m-05-00737-sup1.pdf]

Supporting Information for: Unwarping GISAXS data

Jiliang Liu[a] and Kevin G. Yager [a*]

[a] *Center for Functional Nanomaterials, Brookhaven National Laboratory, Upton, New York 11973 US. E-mail: kyager@bnl.gov*

1. Scattering Features

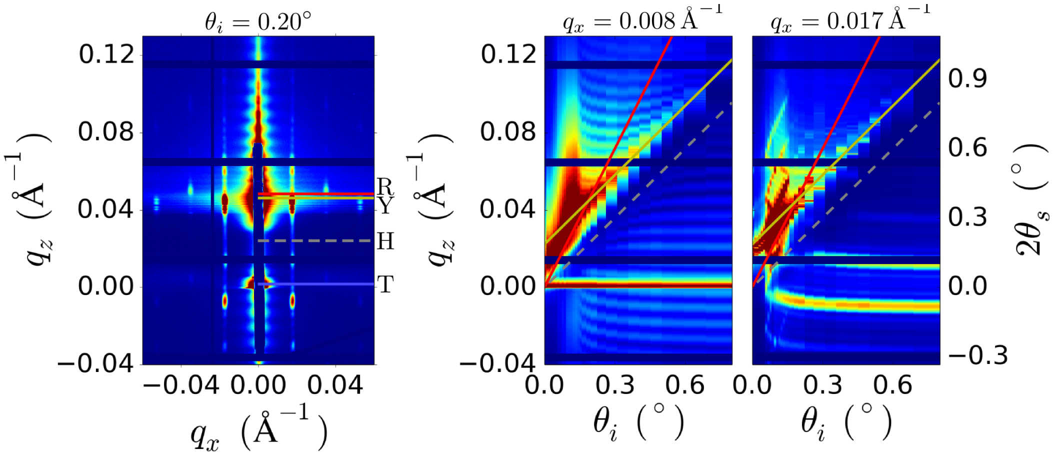

Fig. S1. Experimental example demonstrating the position of various scattering features on the detector image (q_z) as a function of the grazing-incidence angle (θ_i). The reflected beam (R, red) appears at $2\theta_i$, while the horizon (H, gray dashed line) is at half this angle (θ_i). The transmitted beam (T, blue) is roughly at the direct beam position ($q_z = 0$), but is slightly shifted due to refraction. The Yoneda (Y, yellow) appears at θ_c above the horizon (i.e. at total angle $\theta_i + \theta_c$); that is, the Yoneda is defined as the position on the detector when the exit angle matches the critical angle. This detector position probes scattering rays that were traveling nominally within the film plane, and thus probing a larger scattering volume. As can be seen in the experimental data, there is a strong enhancement of all scattering signals along this line (whether viewed along q_x or along θ_i). Of note also is the position of the various scattering peaks, which can be seen to shift as a function of θ_i. Peaks can be seen to 'split' into two branches: one which shifts to higher q_z (and decrease in intensity) as θ_i is increased, and one which decay to a constant value of q_z and intensity as θ_i is increased. The former are peaks from the reflected channel (Rc), where the peaks shift along q_z as the reflected beam moves along the detector (and decrease in intensity since $|R(\theta_i)|$ decreases in value). The latter peaks are from the transmitted channel (Tc), where the peaks are shifted slightly at small θ_i due to refraction of the incident beam.

2. Refraction Correction

Scattering from the sample reciprocal-space at $Q_z = 2k \sin \alpha_s$ (where $2\alpha_s = \alpha_i + \alpha_f$) is measured on the detector at $q_z = 2k \sin \theta_s$ (where $2\theta_s = \theta_i + \theta_f$). The difference between the two values is due to refraction of the incident (i) and scattered (f) beams

(Lu *et al.*, 2013). The refractive shift for the transmitted channel (Tc) is:

$$\begin{aligned}\Delta q_{z,\mathrm{Tc}} &= q_z - Q_z \\ &= q_z - 2k\sin(\alpha_s) \\ &= q_z - 2k\sin\left(\frac{\alpha_i+\alpha_f}{2}\right) \\ &= q_z - 2k\sin\left(\frac{1}{2}\cos^{-1}\left[\frac{\cos\theta_i}{\cos\theta_c}\right] + \frac{1}{2}\cos^{-1}\left[\frac{\cos\theta_f}{\cos\theta_c}\right]\right)\end{aligned} \tag{1}$$

where θ_c is the critical angle for the film-ambient interface ($\cos\theta_c = n_1/n_0$). For the reflection channel (Rc) the scattering is shifted by:

$$\begin{aligned}\Delta q_{z,\mathrm{Rc}} &= q_z - 2k\sin\left(\frac{\alpha_f-\alpha_i}{2}\right) \\ &= q_z - 2k\sin\left(\frac{1}{2}\cos^{-1}\left[\frac{\cos\theta_f}{\cos\theta_c}\right] - \frac{1}{2}\cos^{-1}\left[\frac{\cos\theta_i}{\cos\theta_c}\right]\right)\end{aligned} \tag{2}$$

Notice that the difference is (where small-angle approximations are used repeatedly):

$$\begin{aligned}\Delta q_{z,\mathrm{Rc}} - \Delta q_{z,\mathrm{Tc}} &= -\,2k\sin\left(\frac{1}{2}\cos^{-1}\left[\frac{\cos\theta_f}{\cos\theta_c}\right] - \frac{1}{2}\cos^{-1}\left[\frac{\cos\theta_i}{\cos\theta_c}\right]\right) \\ &\quad + 2k\sin\left(\frac{1}{2}\cos^{-1}\left[\frac{\cos\theta_f}{\cos\theta_c}\right] + \frac{1}{2}\cos^{-1}\left[\frac{\cos\theta_i}{\cos\theta_c}\right]\right) \\ \sin^{-1}\left[\frac{\Delta q_{z,\mathrm{Rc}} - \Delta q_{z,\mathrm{Tc}}}{2k}\right] &\approx -\left(\frac{1}{2}\cos^{-1}\left[\frac{\cos\theta_f}{\cos\theta_c}\right] - \frac{1}{2}\cos^{-1}\left[\frac{\cos\theta_i}{\cos\theta_c}\right]\right) \\ &\quad + \left(\frac{1}{2}\cos^{-1}\left[\frac{\cos\theta_f}{\cos\theta_c}\right] + \frac{1}{2}\cos^{-1}\left[\frac{\cos\theta_i}{\cos\theta_c}\right]\right) \\ 2\sin^{-1}\left[\frac{\Delta q_{z,\mathrm{Rc}} - \Delta q_{z,\mathrm{Tc}}}{2k}\right] &\approx 2\cos^{-1}\left[\frac{\cos\theta_i}{\cos\theta_c}\right] \\ \Delta_{2\theta_s} &\approx 2\sqrt{2\left(1-\left[\frac{\cos\theta_i}{\cos\theta_c}\right]\right)} \\ &\approx 2\sqrt{2\left(1-\left[\frac{1-\theta_i^2/2}{1-\theta_c^2/2}\right]\right)} \\ &\approx 2\sqrt{2-\frac{2}{1-\theta_c^2/2}+\frac{2\theta_i^2/2}{1-\theta_c^2/2}} \\ &\approx \frac{2\theta_i}{1-\theta_c^2/2}\end{aligned} \tag{3}$$

As can be seen, the reflected channel pattern is shifted by the expected amount ($\sim 2\theta_i$) such that it is centered about the specular reflected beam.

3. Reflectivity Calculation

To compute the scattering intensity at the detector for GISAXS experiments, one must compute the relative intensity of scattering terms that are modulated by transmission and reflectivity factors. In particular, we must estimate the intensity of the transmitted and reflected beams that pass through the sample, since the scattering signal is proportional to the photon flux in these channels. We review here the standard method for computing the reflectivity for a thin film (Rauscher *et al.*, 1999; Renaud *et al.*, 2009). First considering the reflectivity at a single interface, the vertical component of the incoming wave vector and the wave vector in the substrate are:

$$\begin{aligned} k_z &= -\sqrt{k^2 - |k_{\|}|^2} \\ \tilde{k}_z &= -\sqrt{n_s^2 k^2 - |k_{\|}|^2} \end{aligned} \tag{4}$$

where $k = 2\pi/\lambda$, $n_s = 1 - \delta - j\beta = \cos\theta_c$ is the refractive index of the substrate ($j = \sqrt{-1}$), and $k_{\|} = k\cos\alpha$ is the in-plane component. The transmission and reflection coefficients can then be written:

$$\begin{aligned} t_s &= \frac{2k_z}{k_z + \tilde{k}_z} \\ r_s &= \frac{k_z - \tilde{k}_z}{k_z + \tilde{k}_z} \end{aligned} \tag{5}$$

For interfaces with roughness $\sigma > 0$, these values can be adjusted to:

$$\begin{aligned} t_\sigma &= t_s e^{-1/2\sigma^2(\tilde{k}_z - k_z)^2} \\ r_\sigma &= r_s e^{-2\sigma^2 k_z \tilde{k}_z} \end{aligned} \tag{6}$$

The coefficients are complex numbers, which can give rise to non-trivial interference effects when multiplied by other factors in the DWBA equation. When generalizing to a multi-layer model, the coefficients for the interface of layer i and $i+1$ become:

$$\begin{aligned} t_{i,i+1} &= \frac{2k_{z,i}}{k_{z,i} + k_{z,i+1}} \\ r_{i,i+1} &= \frac{k_{z,i} - k_{z,i+1}}{k_{z,i} + k_{z,i+1}} \end{aligned} \tag{7}$$

Reflections at different interfaces interfere with each other, with the phase offset being determined by the layer thickness. When layer thickness is taken into account, the coefficients become:

$$\begin{aligned} T_{i,i+1} &= \frac{t_{i,i+1} t_{i+1,i+2} e^{j h_{i+1} k_{i+1}}}{1 + r_{i,i+1} R_{i+1,i+2} e^{2 j h_{i+1} k_{i+1}}} \\ R_{i,i+1} &= \frac{r_{i,i+1} + R_{i+1,i+2} e^{2 j h_{i+1} k_{i+1}}}{1 + r_{i,i+1} R_{i+1,i+2} e^{2 j h_{i+1} k_{i+1}}} \end{aligned} \tag{8}$$

where h_i is the thickness of layer i. The transmission and reflection coefficients (T and R) are complex-valued. However, in the simplified DWBA only their magnitudes $|T|^2$ and $|R|^2$ are required.

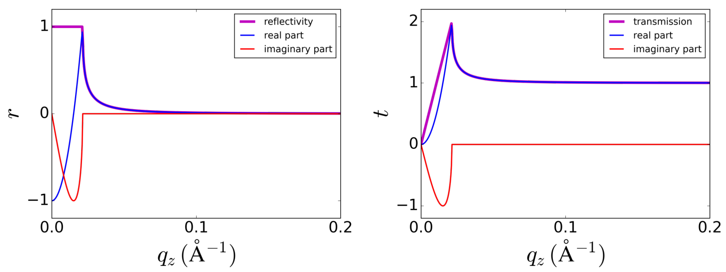

Fig. S2. Examples of the reflection (r) and transmission (t) coefficients for a single interface. These coefficients are complex-valued and may be negative or positive.

4. DWBA Terms

The DWBA equation can be regrouped into the set of independent-terms and the cross-terms:

$$I_d(q_z) = I_{d,\text{ind}}(q_z) + I_{d,\text{cross}}(q_z) \tag{9}$$

Where:

$$
\begin{aligned}
I_{d,\mathrm{ind}}(q_z) &= |T_iT_f|^2|F_{+1}|^2 + |T_iR_f|^2|F_{-2}|^2 + |R_iT_f|^2|F_{+2}|^2 + |R_iR_f|^2|F_{-1}|^2 \\
&= |T_iT_f|^2 I_R(Q_{z1}) + |T_iR_f|^2 I_R(Q_{z2}) + |R_iT_f|^2 I_R(Q_{z2}) + |R_iR_f|^2 I_R(Q_{z1}) \\
I_{d,\mathrm{cross}}(q_z) &= +\, 2|T_i|^2\mathrm{Re}[T_fR_f^*F_{+1}F_{-2}^*] + 2|T_f|^2\mathrm{Re}[T_iR_i^*F_{+1}F_{+2}^*] \\
&\quad + 2|R_i|^2\mathrm{Re}[T_fR_f^*F_{-1}^*F_{+2}] + 2|R_f|^2\mathrm{Re}[T_iR_i^*F_{-1}^*F_{-2}] \\
&\quad + 2\mathrm{Re}[T_iR_i^*T_fR_f^*F_{+1}F_{-1}^*] + 2\mathrm{Re}[T_iR_i^*T_f^*R_fF_{+2}^*F_{-2}]
\end{aligned}
\tag{10}
$$

In general, the independent terms dominate the ultimate scattering, with the cross-terms playing a more minor role. This occurs because the independent terms are constructive: the four terms are all purely-real and strictly positive. The cross-terms, however, may be positive or negative, and have different net phases, causing them to partially or fully cancel one another. An example is shown in Figure S3, where for a particular real-space configuration of material, the corresponding scattering (amplitude and phase components) are shown. The predicted GISAXS when including all DWBA terms (Fig. S3d, black) is only slightly different from the case where the cross-terms are neglected (Fig. S3d, purple). Moreover, in a real GISAXS experiment, the beam averages over an ensemble of statistically-independent volumes, where the nanostructures are randomly different in each sub-volume. In such a case, the overall scattering arises from the (incoherent) sum of the scattering from each sub-volume. The $I_{d,\mathrm{ind}}(q_z)$ component is essentially the same in each sub-volume and is thus retained upon ensemble averaging. The $I_{d,\mathrm{cross}}(q_z)$ component varies randomly in each sub-volume, and thus this contribution becomes less significant after ensemble averaging (Fig. S3e). Overall, this thus justifies neglecting the $I_{d,\mathrm{cross}}(q_z)$ terms, since they are minor contributions compared to the dominant $I_{d,\mathrm{ind}}(q_z)$ component.

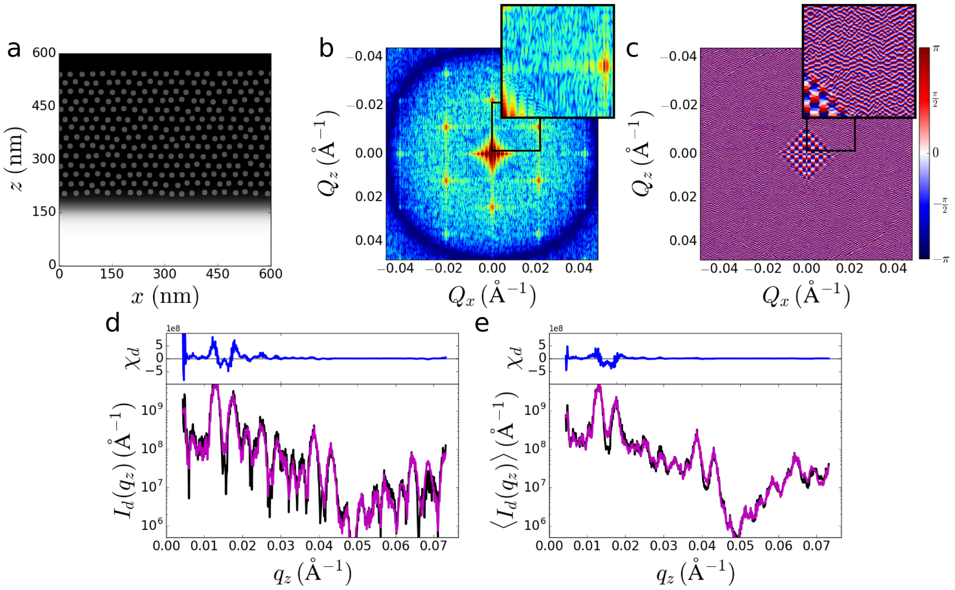

Fig. S3. Relative contributions of terms in a DWBA calculation. (a) An example real-space structure (thin film of hexagonally-packed dots/cylinders) is converted into reciprocal-space scattering in the usual manner (Fourier transform). The complex scattering can be divided into two components: the magnitude of the scattering (b) and the phase of the complex amplitude (c). The square of the magnitude is what is measured experimentally; however the phases of individual scattering components interfere with one another. (d) The GISAXS pattern computed when including all scattering terms (independent and cross) is shown in black. The approximation when using only the independent terms is shown in purple (residuals shown above). As can be seen, neglecting the cross-terms leads to only a minor difference in the prediction. (e) When averaging the GISAXS curves over multiple independent realizations (randomizing particle size and positions), the difference becomes even smaller.

5. Transformation Function

The experimental effect of GISAXS can be thought of in terms of refraction distortion (which maps from Q_z to q_z) and multiple scattering (multiple terms of DWBA). This transformation can be thought of as a single function (which we denote $\mathbb{D}$) that converts the 'true' reciprocal-space scattering $I_R(Q_z)$ into the experimental image

$I_d(q_z)$. Specifically, the transformation for incident angle θ_i is:

$$\begin{aligned} \mathbb{D}[I_R(Q_z),\theta_i] &= \left[|T(\alpha_i)T(\alpha_f)|^2 + |R(\alpha_i)R(\alpha_f)|^2\right] I_R(q_z - \Delta q_{z,\mathrm{Tc}}(\theta_i)) \\ &\quad + \left[|T(\alpha_i)R(\alpha_f)|^2 + |R(\alpha_i)T(\alpha_f)|^2\right] I_R(q_z - \Delta q_{z,\mathrm{Rc}}(\theta_i)) \\ &= |Tc|^2 I_{d,\mathrm{Tc}}(q_z) + |Rc|^2 I_{d,\mathrm{Rc}}(q_z) \\ &= I_d(q_z,\theta_i) \end{aligned} \tag{11}$$

6. Scaling of Fit Error

The mismatch between a candidate reconstruction ($I_{d,\mathrm{test}}(q_z)$) and the experimental GISAXS curve ($I_{d,\mathrm{true}}(q_z)$) is quantified by χ_d^2. We correspondingly denote the mismatch between the reconstructed scattering curve ($I_{R,\mathrm{test}}(Q_z)$) and the true reciprocal-space scattering ($I_{R,\mathrm{true}}(Q_z)$) as χ_R^2. This latter quantity is not known during experiments, but can be approximated by measuring the GTSAXS scattering pattern. In the case of synthetic data, we precisely know $I_{R,\mathrm{test}}(Q_z)$ and can thus evaluate χ_R^2. The initial value of χ_d^2 during the reconstruction of course depends strongly on the quality of the initial guess. Figure S4 shows the decrease of χ_d^2 during iterative reconstruction, for a variety of initial guesses. In all cases, χ_d^2 decreases steadily as the iterative reconstruction proceeds, with χ_R^2 decreasing correspondingly. The strong correlation between these metrics (Figure S5) suggests that fitting by minimizing χ_d^2 will correctly reconstruct the true reciprocal-space (small χ_R^2). Moreover, we find the iterative fitting to be well-behaved in the sense that different choices of initial guess all converge to the same ultimate reconstruction (same ultimate value of χ_d^2). However, as can be seen in Figure S4, the number of iterations required to converge to the ultimate fit varies greatly depending on the initial guess used.

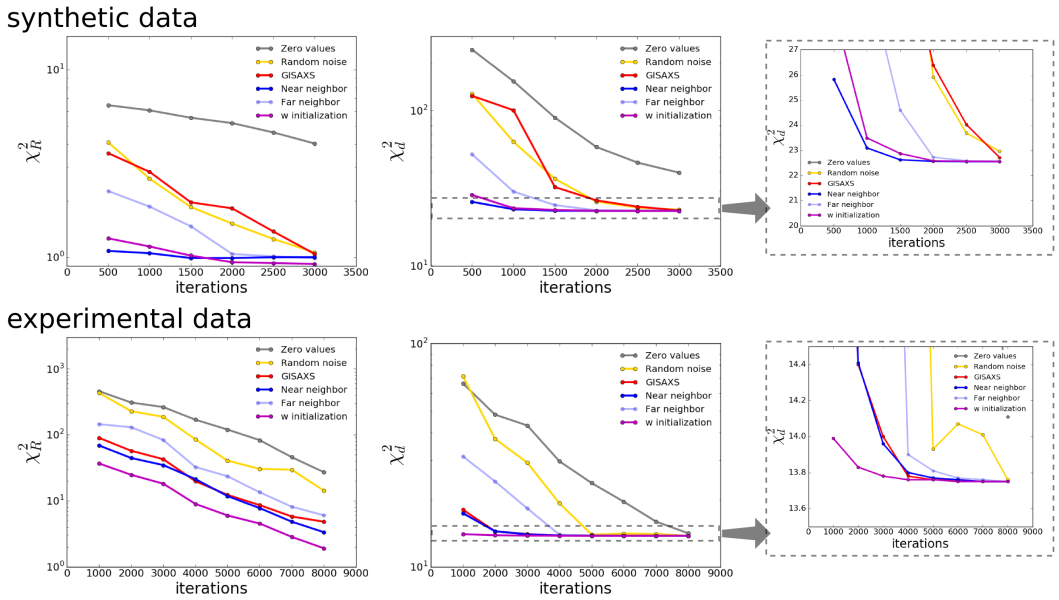

Fig. S4. Scaling of fit error to the experimental data (χ^2_d) and the true scattering (χ^2_R) during iterative reconstruction, for a variety of initial guess strategies. The rightmost plots focus in on the convergence of the most successful strategies. The upper row shows an example of reconstructing synthetic data, where the true reciprocal-space is thus known exactly. The lower row shows an example of fitting experimental data, where we estimate the true reciprocal-space from the best available fit (lowest χ^2_d). The initial guess strategies are: zero values ($I_R(Q_z) = 0$), random noise (initializing with random intensity values), GISAXS (simply taking the experimental GISAXS curve as the initial guess, $I_R(Q_z) = I_d(q_z)$), near neighbor (using a previously-reconstructed nearby column), far neighbor (using a previously-reconstructed column from a very different value of q_x), and w initialization (described below).

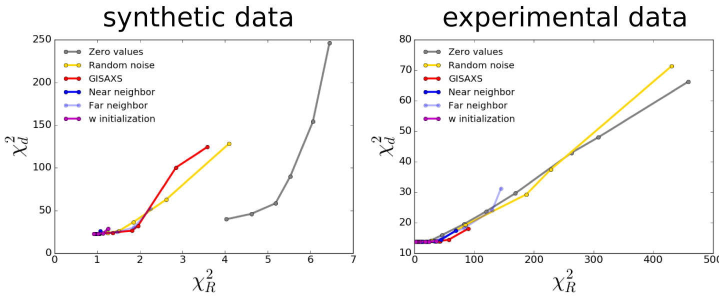

Fig. S5. Correlation between the fit error to the experimental data (χ^2_d), and the (estimated) mismatch to the true reciprocal-space scattering (χ^2_R). The two metrics are highly correlated, with low χ^2_d corresponding to low χ^2_R, which indicates that iterative fitting with minimization of χ^2_d as the target will correctly reconstruct the true reciprocal-space pattern.

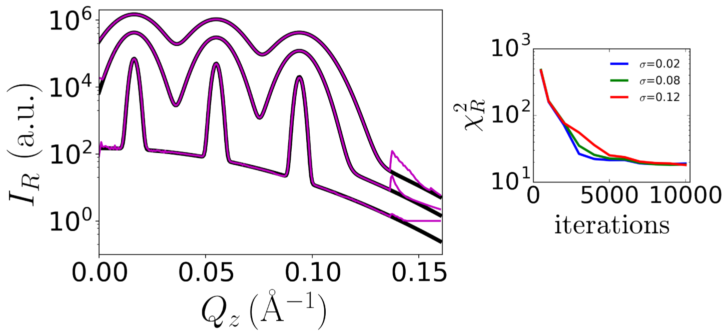

Fig. S6. Examples of the mismatch with true scattering (χ^2_R) when reconstructing different synthetic datasets. In all cases (when reconstructing scattering with sharp or diffuse features), the iterative reconstruction (purple lines) ultimately correctly generates an estimate of the true scattering $I_R(Q_z)$ (black lines), albeit with artifacts near the edges of the available q_z-range. The convergence of the fitting is somewhat better for sharp scattering features (σ denotes the width of the scattering peaks).

7. w initialization

In order to minimize the number of iterations required during reconstruction, the initial guess should be as close to the correct $I_R(Q_z)$ as possible. However, this is a formally underdefined mathematical problem, since the GISAXS signal $I_d(q_z)$ at a particular q_z depends on two different positions (Q_z values) of the $I_R(Q_z)$ curve. Each of these $I_R(Q_z)$ intensities contributes to the signal at two different q_z values. This one-to-many mapping means that there is an underlying correlation between intensities across the full q_z range, with the corresponding system of equations being underdefined. The extreme ends of the $I_d(q_z)$ curve also have contributions from Q_z values outside the experimental range of q_z. Yet, this problem is only weakly underdefined, meaning that it is solvable with relatively modest assumptions. The w initialization strategy (presented in the main text) reformulates the problem from attempting to solve for the two distinct scattering channels ($I_{d,\mathrm{Tc}}(q_z)$ and $I_{d,\mathrm{Rc}}(q_z)$) into attempting to solve for the ratio of these two components, which we denote $w(q_z)$. This method is able to rapidly converge towards a reasonable guess for $I_R(Q_z)$ (Figs. S7–S9).

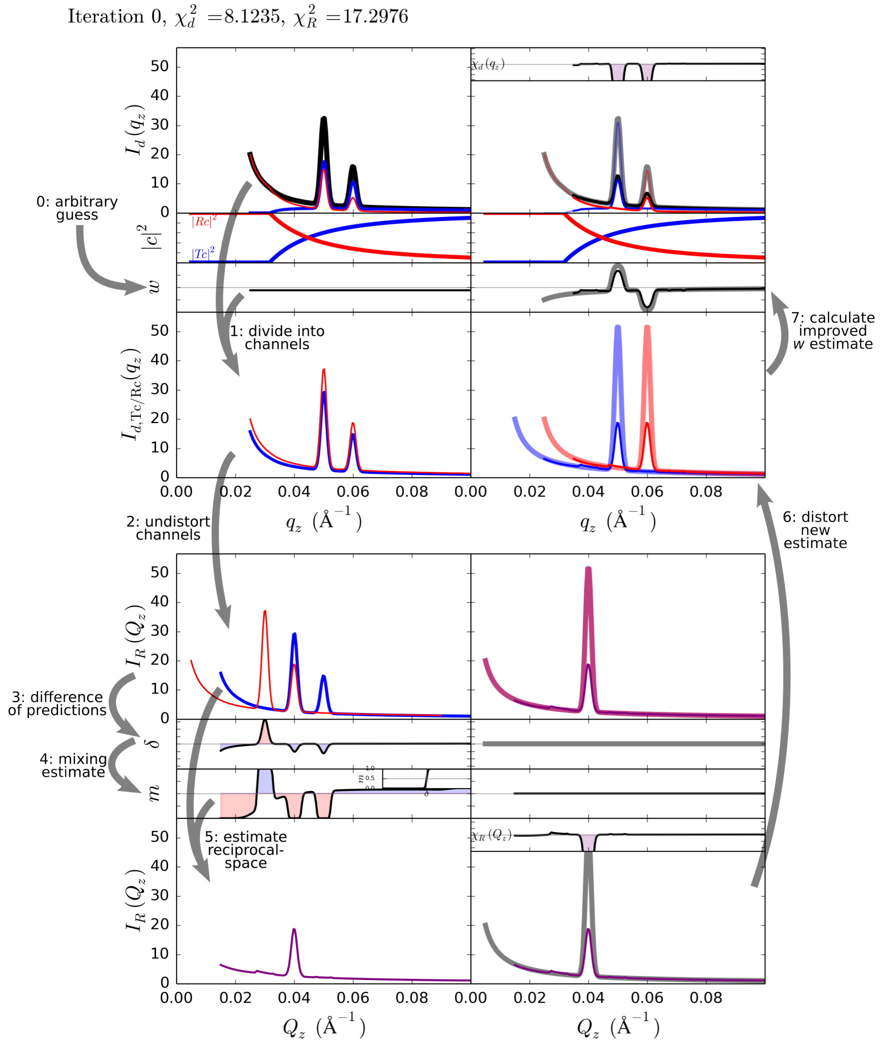

Fig. S7. Example of the first iteration of the w initialization algorithm, which estimates undistorted scattering ($I_R(Q_z)$) from experimental GISAXS data ($I_d(q_z)$). The experimental intensity is first divided into contributions from the transmitted and reflected channels (Tc and Rc) based on known transmission/reflectivity curves ($|Tc|^2$ and $|Rc|^2$) and an arbitrary initial guess for the ratio between the channels (w). The two channel predictions are both undistorted into reciprocal-space, which provides two predictions for the true scattering (which should agree). The difference between these predictions is used to compute an improved estimate for I_R. This I_R estimate can be distorted to yield new estimates for the contributions from the two channels, which provides an improved estimate for w. This new w estimate can be fed back as an improved initial guess. (Thick faded lines show true scattering contributions, while χ shows the corresponding residuals; these are of course not known during reconstruction of experimental data.) By iterating through this procedure, we converge towards a self-consistent prediction for $w(q_z)$ and $I_R(Q_z)$.

Iteration 1, χ_d^2 =0.5869, χ_R^2 =1.3369

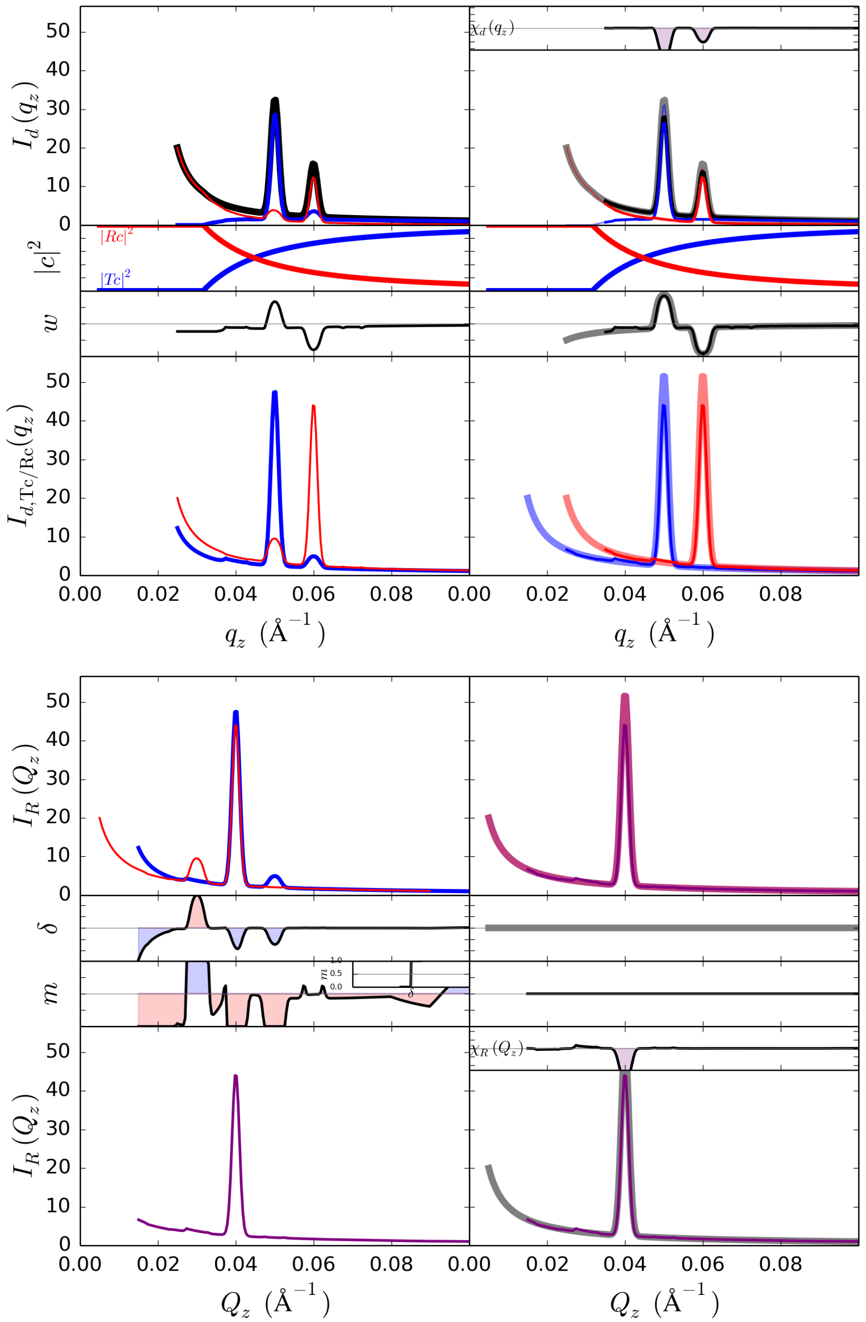

Fig. S8. Example of the second iteration of the w initialization algorithm. Even after only two loops, the method has developed a highly useful estimate for $w(q_z)$.

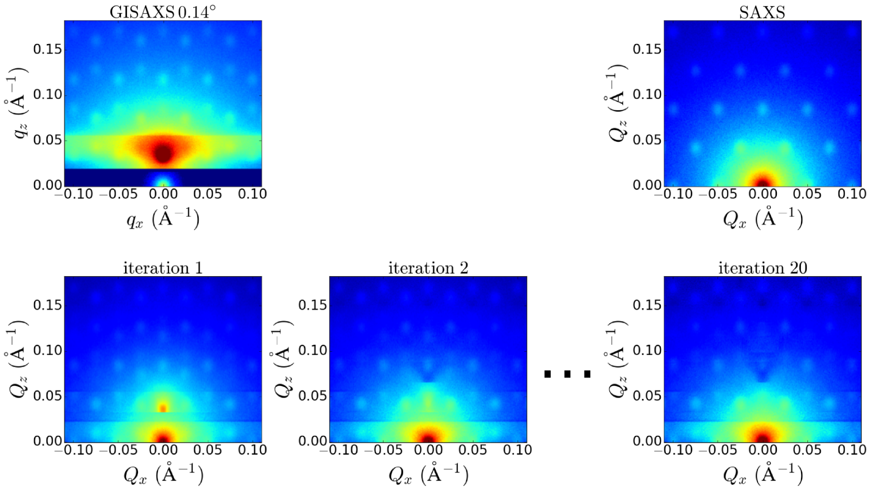

Fig. S9. Example of guessing the form of the undistorted scattering using the w initialization algorithm (synthetic data used for this example). The input GISAXS data is shown on the upper-left. The undistorted scattering pattern (SAXS) is shown on the upper-right. The w initialization rapidly converges (lower row) to a reasonable approximation of the true SAXS pattern, with doubled peaks disappearing. Some intensity artifacts persist; these are eliminated once the initial guess is iteratively reconstructed.

8. Example Reconstructions

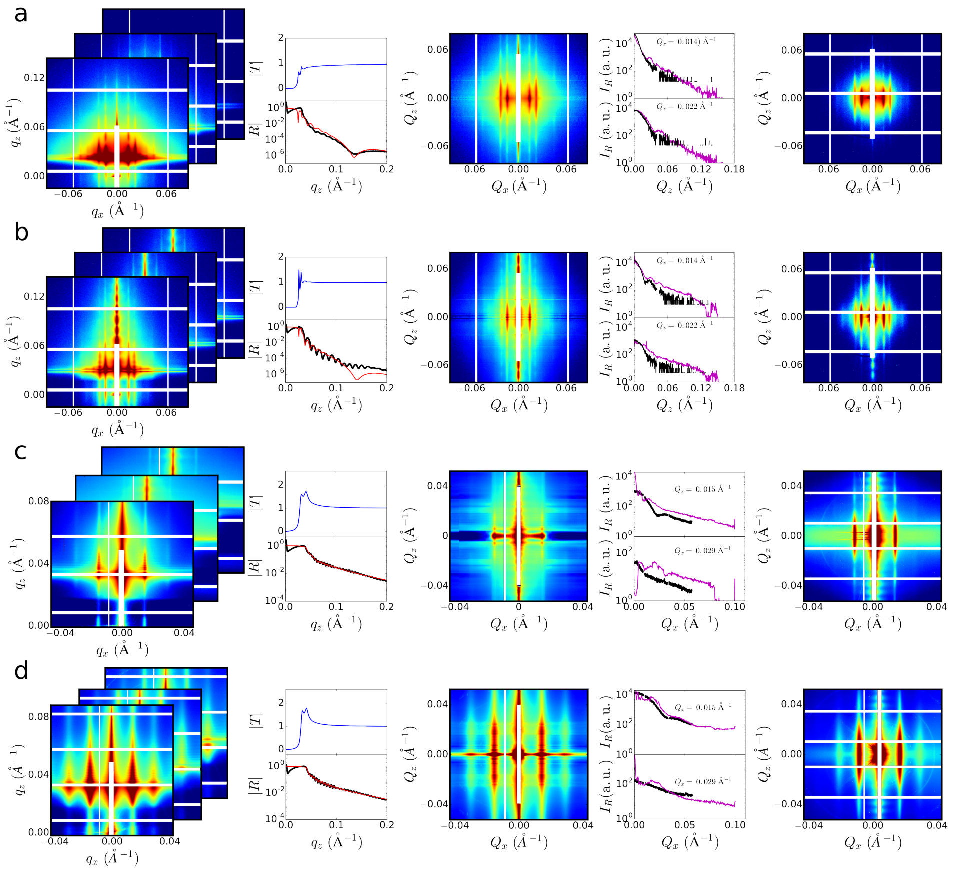

Fig. S10. Example reconstructions based on experimental GISAXS data. Each row shows (from left to right) the set of GISAXS images, the transmission and reflectivity curves (model curves in blue/red; experimentally-measured reflectivity in black), the reciprocal-space reconstruction ($I_R(Q_z)$), select linecuts through the reconstruction (reconstruction in purple, GTSAXS data in black), and the experimental GTSAXS image. The presented data are (a, b) multilayered inorganic nanostructures obtained by layering self-assembling block-copolymer phases, and (c, d) an in-plane hexagonal array of inorganic nano-dots.

9. Reconstruction Sensitivity

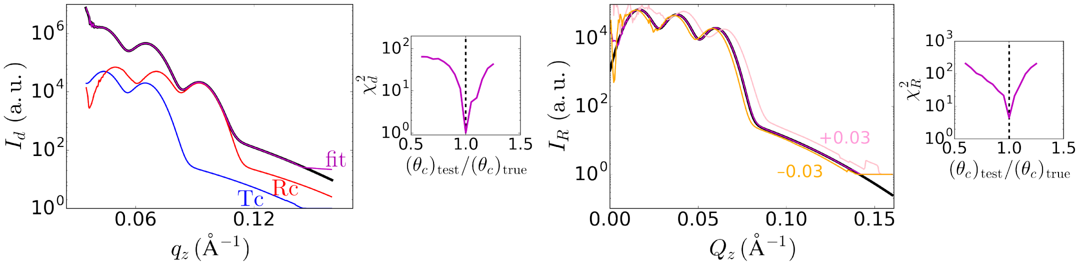

Fig. S11. Sensitivity of reconstruction method to changes in the assumed critical angle, θ_c (based on synthetic data). The experimental GISAXS data (black curve), $I_d(q_z)$ (left) is iteratively fit (purple curve), from which we reconstruct the true reciprocal-space scattering ($I_R(Q_z)$, right); the corresponding transmission channel ($I_{d,\mathrm{Tc}}$) and reflection channel ($I_{d,\mathrm{Rc}}$) components are calculated using the known transmission ($|T|$) and reflectivity ($|R|$) curves. The reconstruction is relativity robust to errors in the assumed critical angle. For a typical dataset (left) with intentional errors introduced into θ_c, the reconstruction becomes correspondingly shifted (right, numbers indicate intentional error in θ_c). The fit-error (χ_d^2) with respect to errors in the angles has a deep minimum at the true value, allowing this value to be iteratively refined also.

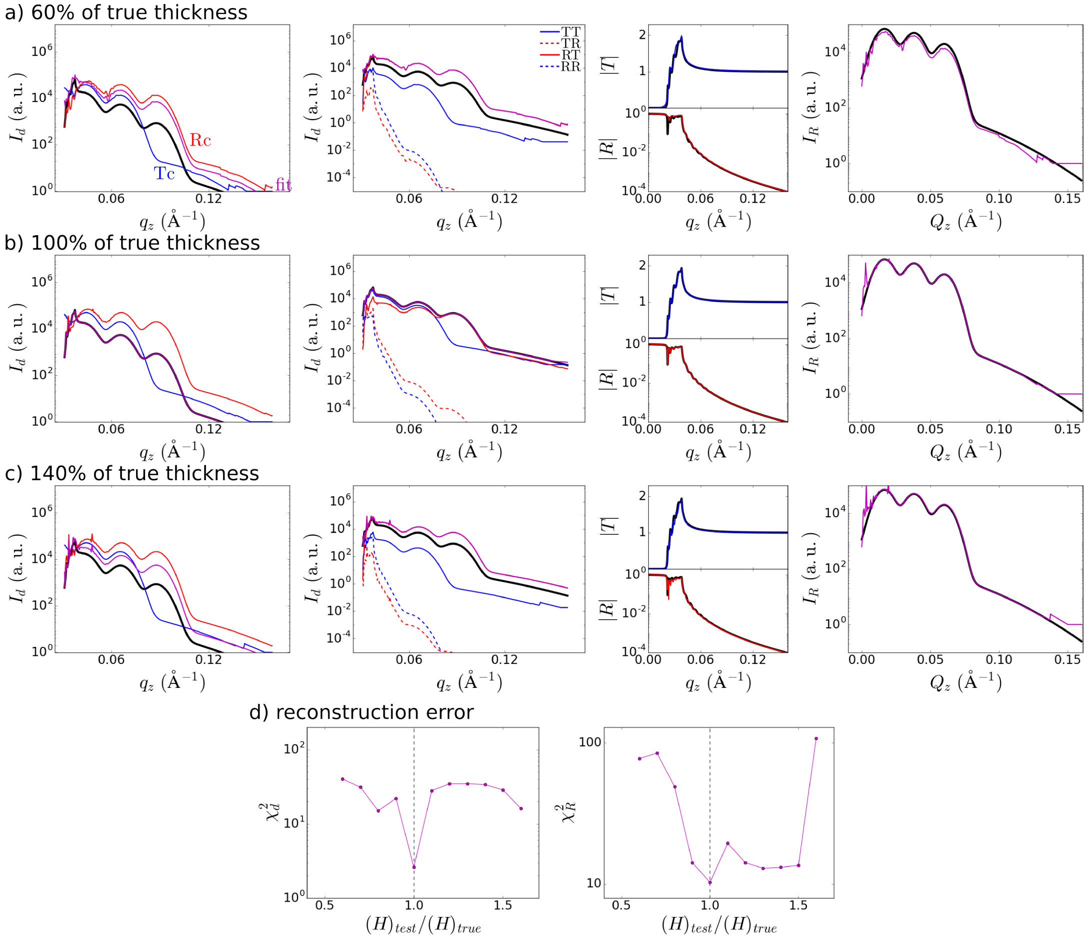

Fig. S12. Sensitivity of reconstruction method to film thickness, H (based on synthetic data). The three rows show three different assumed thicknesses during the reconstruction. The first column shows the reconstruction (purple) compared to the experimental GISAXS (black), with the Tc and Rc contributions shown. The second column shows the 4 DWBA contributions. The third column shows the assumed transmission (blue) and reflectivity (red) curves (true curves in black). The final column compares the reconstruction in reciprocal-space (purple) to the true reciprocal-space (black). Overall, the reconstruction method succeeds even with errors in the assumed thickness. The fit error (d) confirms that the true thickness yields the minimum error.

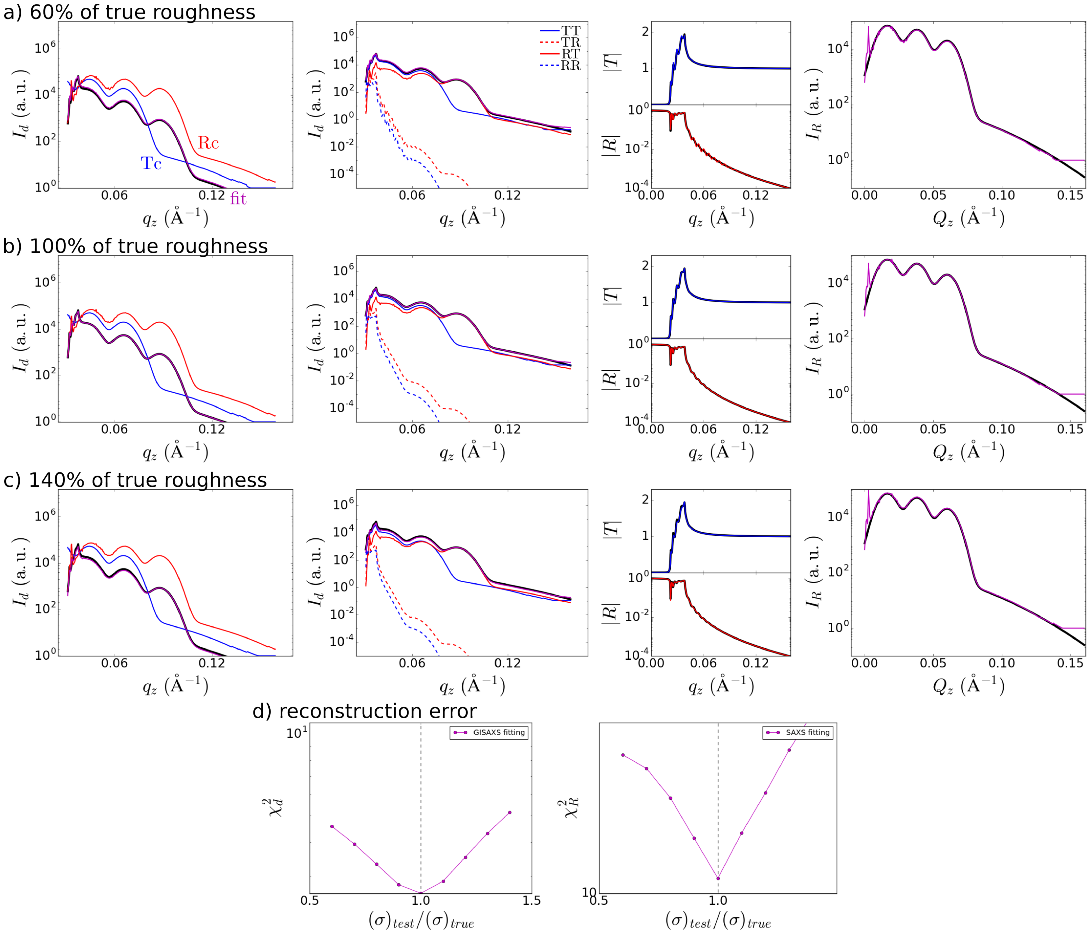

Fig. S13. Sensitivity of reconstruction method to film roughness, σ (based on synthetic data). The three rows show three different assumed roughness values used during the reconstruction. The first column shows the reconstruction (purple) compared to the experimental GISAXS (black), with the Tc and Rc contributions shown. The second column shows the 4 DWBA contributions. The third column shows the assumed transmission (blue) and reflectivity (red) curves (true curves in black). The final column compares the reconstruction in reciprocal-space (purple) to the true reciprocal-space (black). Overall, the reconstruction method is fairly insensitive to errors in the assumed roughness. The corresponding fit error (d) is fairly broad. This insensitivity to roughness makes fitting to obtain this value difficult; but also indicates that exact knowledge of this value is not important for a reasonable reconstruction.

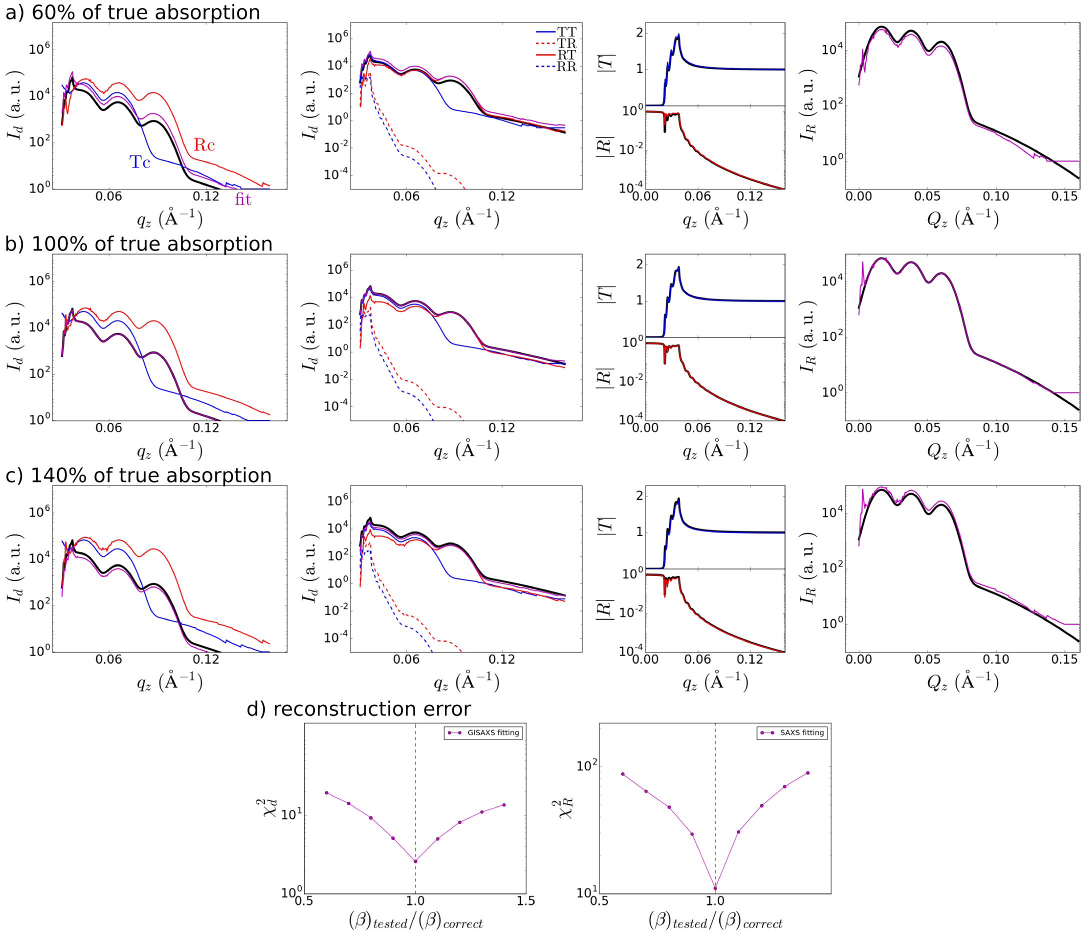

Fig. S14. Sensitivity of reconstruction method to film absorption, β (based on synthetic data). The three rows show three different assumed absorption values used during the reconstruction. The first column shows the reconstruction (purple) compared to the experimental GISAXS (black), with the Tc and Rc contributions shown. The second column shows the 4 DWBA contributions. The third column shows the assumed transmission (blue) and reflectivity (red) curves (true curves in black). The final column compares the reconstruction in reciprocal-space (purple) to the true reciprocal-space (black). Since the reflectivity curve is fairly insensitive to absorption (except below the critical angle), this parameter does not strongly influence the reconstruction. The corresponding fit error (d) confirms that the lowest-error reconstruction occurs at the correct value of absorption.

Acknowledgements

This research used resources of the Center for Functional Nanomaterials, the National Synchrotron Light Source, and the National Synchrotron Light Source II, which are U.S. DOE Office of Science Facilities, operated at Brookhaven National Laboratory under Contract No. DE-SC0012704. Experimental data used in this work was collected at the X9 beamline of NSLS, and the Complex Materials Scattering (CMS, 11-BM) and Soft Matter Interfaces (SMI, 12-ID) beamlines at NSLS-II.

References

Lu, X., Yager, K. G., Johnston, D., Black, C. T. & Ocko, B. M. (2013). *Journal of Applied Crystallography*, **46**(1), 165–172.
URL: *http://scripts.iucr.org/cgi-bin/paper?S0021889812047887*

Rauscher, M., Paniago, R., Metzger, H., Kovats, Z., Domke, J., Peisl, J., Pfannes, H.-D., Schulze, J. & Eisele, I. (1999). *Journal of Applied Physics*, **86**(12), 6763–6769.
URL: *https://aip.scitation.org/doi/abs/10.1063/1.371724*

Renaud, G., Lazzari, R. & Leroy, F. (2009). *Surface Science Reports*, **64**(8), 255–380.
URL: *http://www.sciencedirect.com/science/article/pii/S0167572909000399*
